# Transcriptome analysis of the fish pathogen *Flavobacterium columnare* in biofilm suggests calcium role in pathogenesis

**DOI:** 10.1186/s12866-019-1533-4

**Published:** 2019-07-04

**Authors:** Wenlong Cai, Leonardo De La Fuente, Covadonga R. Arias

**Affiliations:** 1 School of Fisheries, Aquaculture, and Aquatic Sciences, Auburn University, 203 Swingle Hall, Auburn, AL 36849 USA; 2 Department of Entomology and Plant Pathology, Auburn University, Auburn, AL USA

**Keywords:** *Flavobacterium columnare*, Biofilm, Calcium, RNA-seq, Water hardness

## Abstract

**Background:**

*Flavobacterium columnare* is the causative agent of columnaris disease that affects cultured freshwater fishes worldwide. *F. columnare* easily colonizes surfaces by forming biofilm, which helps the pathogen resist antibiotic and disinfectant treatments. Previously, we had shown that increasing concentrations of calcium (Ca^2+^) promoted biofilm formation by *F. columnare*. The objective of this study was to further characterize the role of Ca^2+^ on biofilm formation and to compare the transcriptome profiles of planktonic and biofilm cells.

**Results:**

RNA-Seq analysis was conducted to identify genes that were differentially expressed between the following states: i) planktonic cells in control medium (P), ii) planktonic cells in calcium-enriched medium (P/Ca), and iii) biofilm cells in calcium-enriched medium (B/Ca). Overall, we identified 441 significant (FDR-adjusted *p* < 0.05, fold change > 2) differentially expressed genes (DEGs) between P and B/Ca samples; 112 significant DEGs between P/Ca and B/Ca samples, and 175 significant DEGs between P/Ca and P samples, corresponding to 15.87, 4.03 and 6.30% of the total protein-coding sequences, respectively. The significant DEGs fell into different functional categories including iron acquisition, oxidative stress response, extracellular protein secretion, and respiratory metabolism.

**Conclusions:**

Our results posit Ca^2+^ as a critical signal in regulating bacterial surface adhesion and biofilm formation in *F. columnare*. Living in biofilm elicited a shift in several metabolic pathways that allowed the cells to cope with oxidative stress and nutrient starvation. In addition, Ca^2+^ supplementation induced the expression of putative virulence factors in *F. columnare*, such as extracellular protein secretion and iron acquisition.

**Electronic supplementary material:**

The online version of this article (10.1186/s12866-019-1533-4) contains supplementary material, which is available to authorized users.

## Background

*Flavobacterium columnare* is a Gram negative bacterium that is the causative agent of columnaris disease in fish. This bacterial fish pathogen causes great economic losses in key aquaculture species worldwide such as channel catfish, tilapia, and trout [1]. *Flavobacterium columnare* is considered to be ubiquitous in freshwater environments including fish farms. Recurrent columnaris outbreaks are common in farms and eradication is extremely difficult. Columnaris disease is transmitted horizontally by fish-to-fish contact and asymptomatic fish can easily vector the pathogen into a farm. Although columnaris disease can be transmitted through water, cell counts of *F. columnare* in water are typically very low, even during active outbreaks [2]. Once fish are removed from a system, *F. columnare* quickly disappears from the water column suggesting that, in the aquatic environment, the planktonic state of *F. columnare* is transient [2]. Conversely, *F. columnare* colonizes natural and manmade materials quickly and remains viable, and infective, in biofilms even when fish host are not available [3].

Biofilms can be defined as bacterial communities that are attached to solid surface and covered with exopolysaccharides. Living in a biofilm offers aquatic bacteria many advantages over the alternative planktonic stage including a better adaptation to nutrient deprivation, and increased resistance to stressors such as desiccation and antimicrobial compounds [4]. Biofilm development requires several key steps, i.e., transport and attachment of planktonic bacteria onto a surface, cell proliferation, formation of microcolonies, and dispersion of daughter cells into the water column [5]. In vivo studies by Straus et al. (2015) demonstrated that higher calcium (Ca^2+^) concentrations in rearing tanks water increased *F. columnare* attachment to gills and subsequent host colonization [6]. Columnaris disease is primarily an epithelial disease that causes necrotic skin and gill lesions [1]. As *F. columnare* colonizes the host, whitish plaques often appear along the fish body that exhibit yellow borders due to the masses of pigmented *F. columnare*. In a sense, columnaris disease can be considered a biofilm infection that invades the fish from the outside in [7]. Previous studies have shown that Ca^2+^ promotes binding to surfaces and biofilm formation by *F. columnare* [3, 8] and appears to enhance virulence in fish, however, the mechanisms by which calcium regulates these processes are unknown.

The objectives of this study were: i) to elucidate the differential transcriptional regulation between planktonic and biofilm states of growth, and ii) to determine the effect of Ca^2+^ in the transcriptional regulation of planktonic cells. This transcriptional analysis increased our understanding of the effect of environmental stimuli (Ca^2+^) and subsequent cell responses that drive successful biofilm colonization.

## Results

### Biofilm formation under increased [Ca^2+^]

**Fig. 1:**
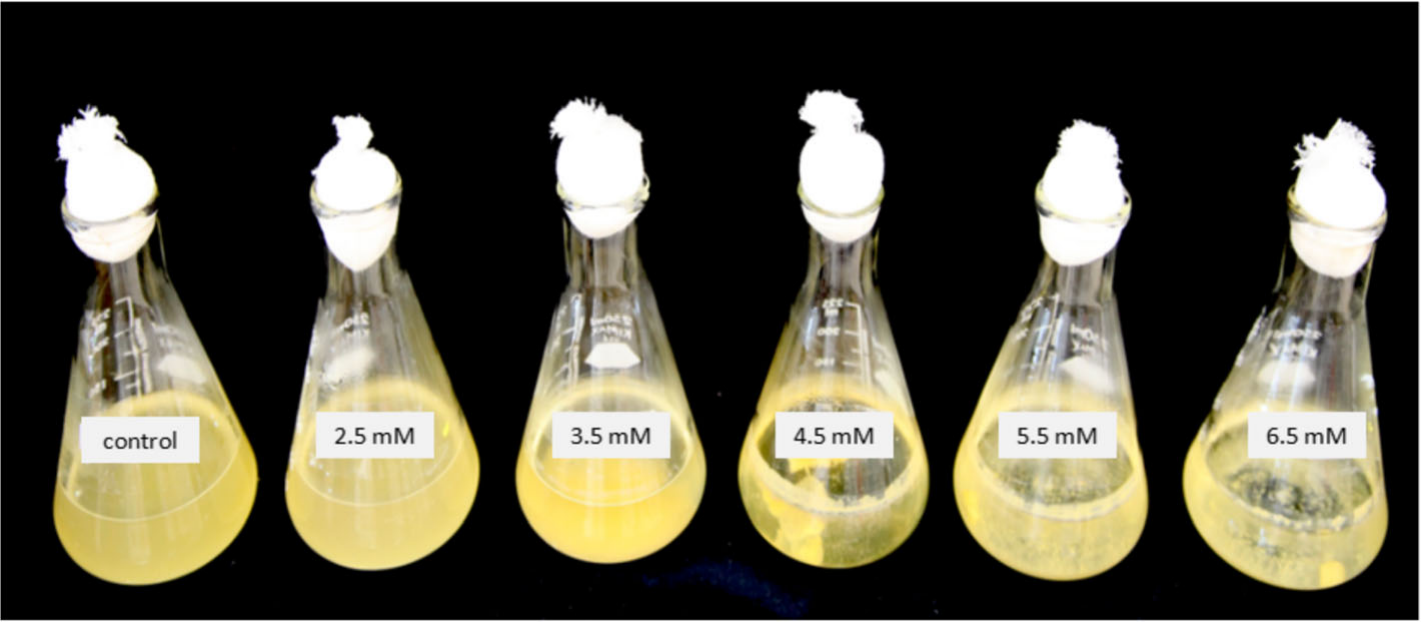
Biofilm formation of *F. columnare* in Erlenmeyer flasks after 48 h inoculation under different calcium concentrations. Visually, there was a positive correlation between Ca^2+^ and biofilm formation in the flasks and a negative correlation between Ca^2+^ and planktonic cells. There was no observable biofilm in control medium while no turbidity was seen at the highest Ca^2+^ concentration

Calcium supplementation promoted biofilm formation by *F. columnare* on the wall of flasks. Without Ca^2+^ supplementation, *F. columnare* cells remained planktonic and there was no observable biofilm in the flasks (Fig. 1). For this reason, we were not able to obtain enough RNA for whole transcriptome analysis of biofilms growing in control medium. At the other end of the spectrum, at 6.5 mM [Ca^2+^], cells grew in large clusters that quickly precipitated to the bottom after shaking ceased and there was no visible biofilm on the glass walls. Moreover, at 6.5 mM [Ca^2+^], there was no turbidity in the medium due to the absence of planktonic cells. Based on those observations, 4.5 mM [Ca^2+^] was deemed as the best concentration to use for RNA-Seq analysis since it produced sufficient biofilm on the walls of the flask while enough planktonic cells remained in culture.

### Quantitative analysis of global gene expression

**Fig. 2:**
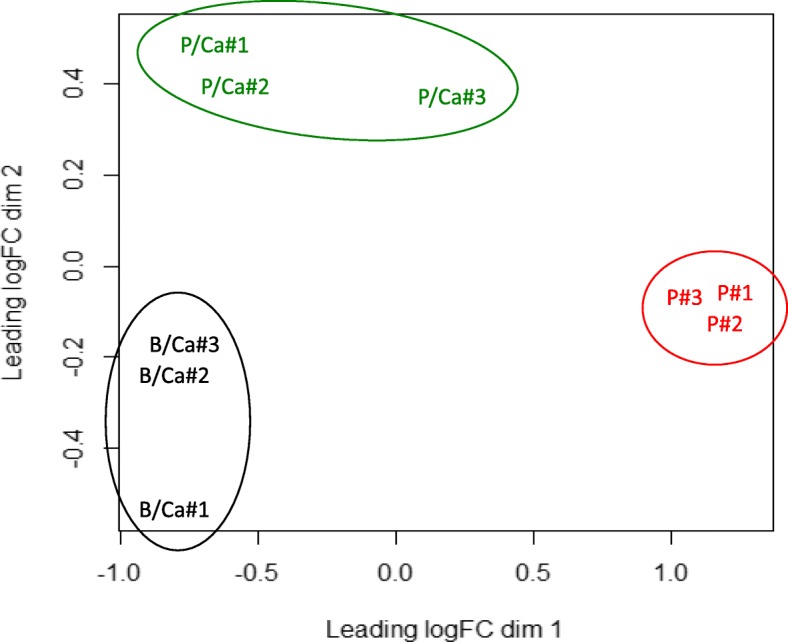
Principal Component Analysis of biological replicates after mapping to reference sequence *Flavobacterium columnare* 94–081. P, planktonic samples in control medium; P/Ca, Ca-supplemented medium (4.5 mM); B/Ca, biofilm samples in Ca-supplemented medium (4.5 mM)

The mapped reads, using *F. columnare* 94–081 as reference genome, ranged from 12.5 M to 22 M with an average of 15.4 M per sample (Additional file 2: Figure S1). Principal component analysis (PCA) was performed on the raw RNA-seq reads that mapped to the reference genome to evaluate repeatability. The PCA plots showed that the biological replicates were well aggregated (Fig. 2). On PC1, there was a clear differentiation between the transcripts in planktonic cells with and without calcium amendment, while the differentiation between the biofilm cells under calcium amendment and planktonic cells under calcium amendment was observed on PC2.

**Fig. 3:**
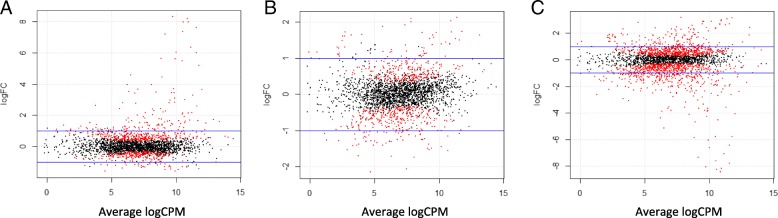
PlotSmear graph of the samples. Panel **a** Genes expressed in planktonic cells cultured in Ca-supplemented medium (up) vs planktonic cells cultured in control medium (down). Panel **b** Genes expressed in planktonic cells cultured in Ca-supplemented medium (up) vs biofilm in Ca-supplemented medium (down). Panel **c** Genes expressed in planktonic cells cultured in control medium (up) vs biofilm in Ca-supplemented medium (down). The red dots represent DEGs with *p* > 0.05, and the blue lines set threshold with a fold change > 2

Figure 3 plots genes exhibiting significant expression changes (fold change ≥2; FDR-adjusted *p* < 0.05) among the three pair-wise comparison groups. Overall, we identified 175 DEGs (135 upregulated, 40 downregulated) between planktonic-control (P) and planktonic-4.5 mM calcium (P/Ca) groups (Fig. 3a); 112 DEGs (61 upregulated, 51 downregulated) between P/Ca and biofilm-4.5 mM calcium (B/Ca) groups (Fig. 3b), and 441 DEGs (286 upregulated, 155 downregulated) between P and B/Ca groups (Fig. 3c). These genes represent 6.30, 4.03, and 15.87% of the total protein-coding genes, respectively. A heatmap of the top 50 differentially expressed genes expression profiles was produced using Blast2Go software v 4.0. Differentially regulated genes were compared among the three treatment groups. Gene expression profiles of P and P/Ca samples were more similar to each other than those exhibited by B/Ca samples (Additional file 2: Figure S2).

### DEGs identified between biofilm and planktonic cells under equal [Ca^2+^]

**Fig. 4:**
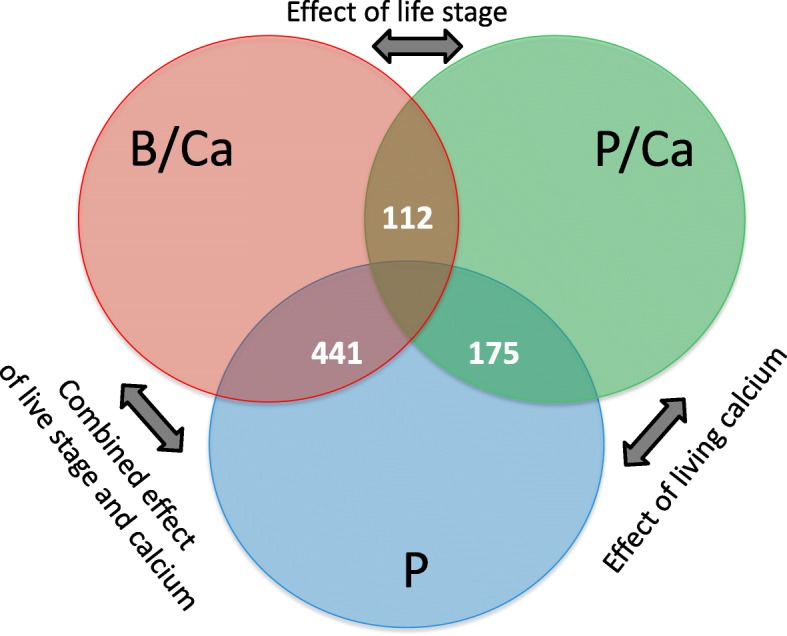
Venn diagram of the number of significant DEGs among the different biological groups. Three comparison were made: B/Ca:P/Ca; B/Ca:P: P/Ca:P

One hundred and twelve genes were differentially expressed due to the effect of biofilm as identified between B/Ca and P/Ca (Fig. 4). The top three upregulated DEGs in biofilm were catalase (WP_060381974; log2 Fc = 2.16), DNA starvation protect protein (WP_060382448; log2 Fc = 2.08), and a hypothetical protein (WP_060382447; log2 Fc = 1.75) that contains an EF-hand motif with calcium-binding function (Table 1). Other upregulated DEGs in biofilm fell into several functional categories including oxidative stress response, transcriptional regulators, TonB-dependent receptor, and carbohydrate biosynthesis (Table 1 and Additional file 1). In addition, two of upregulated genes in biofilm included a ribosome inactivating protein gene (WP_060383199) that mediates the negative regulation of translation, and an abortive phage infection protein gene (WP_060381920) that leads to programmed death of cells for self-protection [9]. Gene ontology analysis indicated upregulated genes were mostly categorized as integral components of membranes (GO: 0016021; Additional file 1: Figure S3). The top downregulated DGEs in biofilm were associated with aerobic respiration metabolism, type IX secretion system (T9SS) and protein biosynthesis (Table 1).

**Table 1:** Selected genes in key functional categories differentially expressed in biofilm compared to planktonic cells (both cultured in 4.5 mM [Ca^2+^]). Genes were selected based on fold change and their functional relevance

Functional group	RefSeq	Product	Fold change (log2)	FDR (adjusted *p* value)
EPS/LPS biosynthesis				
	WP_060381967.1	1,4-polygalactosaminidase	1.72	1.1E-19
	WP_060381836.1	UDP-N-acetylmuramate--alanine ligase	1.24	4.1E-05
	WP_060381978.1	SAM-dependent methyltransferase	1.04	3.8E-07
	WP_060382475.1	Glycosyltransferase	− 1.99	9.3E-03
Amino acid metabolism				
	WP_060383852.1	Branched-chain amino acid aminotransferase	−1.53	3.52E-16
	WP_060382313.1	Anthranilate synthase	−1.35	2.71E-10
	WP_060382314.1	Anthranilate phosphoribosyltransferase	−1.03	1.18E-06
	WP_060382792.1	Glycyl aminopeptidase M61	1.11	2.2E-11
	WP_060382658.1	Von 25illebrand factore type protein	1.54	5.8E-10
T9SS				
	WP_060383582.1	T9SS C-terminal target domain-containing protein	−1.53	3.52E-16
	WP_060383402.1	T9SS C-terminal target domain-containing protein	−1.13	3.07E-05
Nutrient limitation				
	WP_060381920.1	Abortive phage infection protein	1.17	5.0E-08
	WP_060383199.1	Ribosome inactivating protein	1.30	9.27E-10
	WP_060381977.1	Protein-tryosine-phosphatase	1.04	1.39E-09
	WP_060382448.1	DNA starvation protect protein under stressful or poor nutriention conditions	2.08	8.3E-23
Regulatory functions				
	WP_060382614.1	AraC family transcriptional regulator	1.71	2.9E-11
	WP_060381931.1	Transcriptional regulator	1.31	5.4E-08
	WP_060381980.1	ArsR family iron regulation transcriptional regulator	1.54	4.7E-4
Oxidative stress response				
	WP_060381974.1	Catalase	2.16	8.71E-10
	WP_060381975.1	Alkyl hydroperoxide reductase	1.50	8.29E-15
	WP_060382235.1	Cytochrome c peroxidase	1.41	1.31E-10
	WP_014164670.1	Thiol reductase thioredoxin	1.03	2.35E-09
Respiratory metabolism				
	WP_060381919.1	O-succinylbenzoic acid CoA ligase	1.11	7.3E-03
	WP_060382480.1	Cytochrome C oxidase subunit III	−2.12	7.45E-06
	WP_060382479.1	Cytochrome c oxidase accessory protein CcoG	−2.03	2.9E-06
	WP_060382481.1	Cytochrome C oxidase subunit IV	−1.74	2.3E-05
	WP_060382478.1	Cytochrome C oxidase Cbb3	−1.74	1.94E-03
	WP_060381577.1	Cytochrome c oxidase subunit I	− 1.50	1.7E-02
	WP_060381570.1	Cytochrome C	−1.39	3.7E-04
	WP_060381576.1	Cytochrome C oxidase subunit II	−1.39	2.6E-02
	WP_060381561.1	NADH dehydrogenase	−1.27	6.5E-03
	WP_060381560.1	NADH oxidoreductase (quinone) subunit F	−1.18	6.1E-03
	WP_060381566.1	NADH-quinone oxidoreductase subunit L	−1.07	1.7E-03
	WP_060381562.1	NADH:ubiquinone oxidoreductase subunit H	−1.06	1.1E-02
	WP_014164161.1	NADH-quinone oxidoreductase subunit I	−1.00	9.1E-03

### DEGs between planktonic cells in calcium-supplemented medium and control medium

There were 175 DEGs between P/Ca and P comparison (Fig. 4). We found more upregulated genes than downregulated genes (135 vs 40) in planktonic cells cultured in calcium-supplemented medium, indicating that Ca^2+^ activated several metabolic pathways in the cells. Upregulated genes fell into several functional groups including siderophore synthesis, calcium homeostasis, iron acquisition, and genes coding for proteins involved in secretion pathways such as T9SS, flagellar motor protein, and aquaporin (Table 2). On the other side, most of the downregulated genes were associated with aerobic respiration (Additional file 1).

**Table 2:** Selected genes in key functional categories differentially expressed in planktonic cells cultured in 4.5 mM [Ca2+] compared to planktonic cells cultured in control medium

Functional group	RefSeq	Product	Fold change (log2)	FDR (adjusted *p* value)
Siderophore synthesis				
	WP_060383187.1	Siderophore alcaligin biosynthesis protein	8.20	4.1E-41
	WP_060383190.1	LucA/lucC family siderophore biosynthesis protein	5.64	1.7E-44
	WP_060383888.1	Siderophore biosynthesis protein	7.21	6.4E-61
Iron transfer				
	WP_060383657.1	Iron transporter	2.45	3.7E-13
	WP_060383656.1	Ferrous iron transport protein B	1.53	3.2E-09
	WP_060381373.1	NifU family protein (iron-sulfur cluster binding)	1.19	4.7E-10
T9SS secretion				
	WP_060382679.1	T9SS C-terminal target domain-containing protein	7.61	8.0E-43
	WP_060383582.1	T9SS C-terminal target domain-containing protein	1.58	2.8E-05
	WP_060383035.1	T9SS C-terminal target domain-containing protein	1.65	6.6E-10
TonB-dependent receptor				
	WP_060383861.1	TonB-dependent receptor	6.03	6.9E-40
	WP_060383182.1	TonB-dependent receptor	6.01	4.4E-49
	WP_060383101.1	TonB-dependent receptor	5.18	3.7E-33
	WP_060382917.1	TonB-dependent receptor	−1.22	5.7E-04
	WP_060382921.1	TonB-dependent receptor	−1.59	4.4E-02
Calcium homeostasis				
	WP_060383353.1	ABC transporter permease	1.56	1.9E-11
	WP_060383434.1	Outmembrane efflux protein	1.46	2.3E-08
	WP_060383433.1	Efflux transporter periplasmic adaptor	1.21	1.7E-07
Integral membrane component				
	WP_060381689.1	Flagellar motor protein MotA	1.47	2.9E-06
	WP_060383854.1	Aquaporin	1.69	1.9E-11
Respiration system				
	WP_060381562.1	NADH:ubiquinone oxidoreductase subunit H	−1.01	1.4E-02
	WP_060381556.1	NADH dehydrogenase	−1.02	2.4E-03
	WP_060381560.1	NADH oxidoreductase (quinone) subunit F	−1.12	9.2E-03
	WP_060381555.1	NADH-quinone oxidoreductase subunit A	−1.12	5.3E-03
	WP_060381577.1	Cytochrome c oxidase subunit I	−1.20	5.4E-02
	WP_060381576.1	Cytochrome C oxidase subunit II	−1.23	3.5E-02
	WP_060381574.1	Cytochrome C	−1.30	3.2E-02
	WP_060382919.1	Cytochrome-c peroxidase	−1.31	8.7E-03
	WP_060381571.1	Quinol:cytochrome C oxidoreductase	−1.41	3.2E-02
	WP_060381575.1	Quinol:cytochrome C oxidoreductase	−1.32	2.9E-02
Gliding motility	WP_060383259.1	Gliding motility protein GldN	−1.01	1.5E-02
Quorum sensing	WP_060383185.1	LuxR family transcriptional regulator	3.94	2.8E-29

### Oxidative stress response in biofilm

A number of antioxidative genes (e.g. catalase, *Ahp*C, cytochrome c peroxide, and thiol reductase thioredoxin) were identified in response to oxidative stress in biofilm-associated growth (Table 1). Catalase (WP_060381974.1; log2Fc = 2.16) was the most upregulated protein-coding gene in biofilm samples when compared to planktonic cells of the same [Ca^2+^]. This enzyme breaks down hydrogen peroxide into water and oxygen and its production might increase in response to the elevated reactive oxygen species (ROS) in biofilm. Alkyl hydroperoxide reductase (AhpC; WP_060381975.1; log2Fc = 1.5) is another upregulated antioxidative product in this category, which is the primary scavenger of endogenous hydrogen peroxide in bacterial cells.

### Aerobic respiration is greatly limited in biofilm

Among the top 27 downregulated gene in biofilm, up to 11 genes were associated with cytochrome c respiratory electron transfer (Additional file 1: Table S1), including cytochrome c oxidase (WP_060382480.1), cytochrome c peroxidase (WP_060381419.1), and NADH-ubiquinone reductase (WP_060381562.1). In addition, the gene that codes for the o-succinylbenzoate CoA ligase (WP_060381919.1), an enzyme involved in menaquinone (vitamin K_2_) biosynthesis was highly upregulated in biofilm samples. Interestingly, menaquinone is the major electron carrier during anaerobic metabolism and can use various electron acceptors.

### Calcium supplementation promotes iron acquisition

In addition to the cytoplasmic membrane (CM), common to all cells, Gram-negative bacteria possess an outer membrane (OM), which hinders the uptake of essential nutrients. In bacterial cells, iron uptake is conveyed by siderophore transport system. In this study, siderophore biosynthesis protein (WP_060383187.1) was the second (logFc = 8.2) highest upregulated protein in the planktonic cells under calcium supplementation. We identified a number of protein-coding genes coding for iron acquisition and transport system (Table 2). These include the siderophore biosynthesis, TonB dependent transport system, iron transporter (WP_060383657), ferrous iron transport protein (WP_060383656), and NifU family protein containing iron-sulphur cluster (WP_060381373). There were up to 3 siderophore biosynthesis genes (log2Fc = 5.6–8.2) and 5 TonB dependent receptor genes associated with siderophore transportation (log2Fc = 1.1–6.1) were upregulated totally in the calcium-supplemented medium (Table 2).

### Calcium supplementation promotes protein secretion by T9SS

Type IX secretion system (T9SS) is an outer membrane protein secretion system. The common T9SS substrates include proteins involved in gliding and extracellular proteases. Precursor of the T9SS cargo protein contains a conserved C-terminal domain (CTD) during the cargo’s translocation across the outer membrane. In our study, multiple genes encoding T9SS C-terminal target domain-containing proteins (WP_060382679.1; WP_0603833582.1; WP_060383035.1; WP_060383402.1) were identified in the upregulated genes in the calcium-supplemented samples (Table 2).

### Validation of RNA-seq profiles by qPCR

**Fig. 5:**
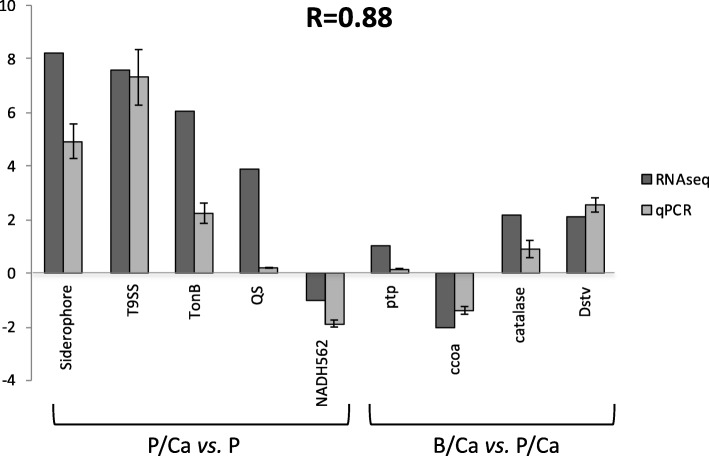
Comparison of relative fold change between RNA-seq and qPCR results using DEGs in P/Ca and P, and B/Ca and P/Ca. Protein coding sequences abbreviations: Siderophore alcaligin biosynthesis protein (WP_060383187), siderophore; T9SS secretion (WP_060382679), T9SS; Ton-B dependent receptor (WP_060383861), TonB; Quorum sensing (WP_060383185), QS; NADH subunit H (WP_060381562), NADH562; Protein-tyrosine-phosphate (WP_060381977), ptp; Cytochrome c oxidase accessory (WP_060382479), ccoa; Catalase (WP_060381974), catalase; DNA starvation protect protein (WP_060382448), Dstv

In order to validate the differentially expressed genes identified by RNA-seq, 10 genes from various categories were selected for qPCR confirmation. Fold changes from qPCR were compared with the RNA-seq express analysis results. As shown in Fig. 5, qPCR results were significantly correlated with the RNA-seq results (correlation coefficient 0.88), indicating a consistency with the transcriptional expression analysis.

## Discussion

Calcium concentration contributes to water quality in the form of water hardness. In general, the effect of calcium to bacterial biofilm formation has been shown to be species-specific. A previous study in our lab showed that the addition of calcium strongly promoted biofilm formation in *F. columnare* [3]. Similar results have been found in *Pseudomonas aeruginosa* [10], *Sinorhizobium meliloti* [11], *Xylella fastidiosa* [12], *Enterobacter cloacae* [13], *Aeromonas hydrophila* [8], and *Enterococus faecalis* [14]. On the contrary, the addition of calcium suppresses biofilm formation in *Vibrio cholerae* [15] and *Staphylococcus aureus* [16].

We have previously established that *F. columnare*, a strictly aerobic bacterium, favors the production of biofilm at the air/liquid interface on glass slides [3] and on other substrates [8] where oxygen is abundant. The consumption of molecular oxygen leads to the accumulation of reactive oxygen species (ROS), potentially leading to oxidative stress [17]. Interestingly, oxidative burst has been proposed as a signal that induces bacteria to form biofilms in order to protect themselves against adverse environmental conditions [18]. In addition, this mechanism seems to be conserved among both Gram-positive and Gram-negative bacterial species. For example, *P. aeruginosa* increased the synthesis of the exopolysaccharide (EPS) alginate when it was exposed to hydrogen peroxide [19]. In the presence of oxidative stress, increased production of polysaccharides was observed in *Azotobacter vinelandii* [20] and *Bacillus subtilis* [21] biofilm matrices. Although oxidative stress has been broadly observed during biofilm development, it is still unclear whether oxidative stress triggered the biofilm formation or oxidative was the result of metabolism during biofilm development.

In this study, we found that genes involved in aerobic respiration were greatly downregulated in biofilm samples, which indicated a reduced aerobic metabolism in biofilm. Noteworthy was the upregulation of o-succinylbenzoate CoA ligase in biofilm samples, which catalyzes the menaquinone biosynthesis. Menaquinone plays an essential role in several anaerobic electron transport systems as it is the major electron carrier during anaerobic metabolism and can use various electron acceptors [22]. This process of anaerobic respiration allows bacteria to generate the energy required to survive under oxygen-limited conditions. This observation was interesting because *F. columnare* has been regarded as a strictly aerobic bacterium. Although anaerobic respiration in *F. columnare* has not yet been reported, bioinformatics analysis showed that, indeed, the *F. columnare* genome encodes for enzymes that are involved in anaerobic metabolism, such as denitrification that could, theoretically, allow the bacterium to remain metabolically active under anaerobic conditions. This in silico prediction was supported by the fact that high levels of *F. columnare* cells have been detected in anaerobic sediments of catfish ponds by metagenomic analysis [23].

Iron is a co-factor of many enzymes that plays a crucial role in diverse physiological processes such as DNA replication, transcription and central metabolism [24]. Iron is required by virtually all bacterial pathogens and vertebrates, therefore iron acquisition becomes a critical survival strategy during host-pathogen interactions. As a result, bacteria must elaborate Fe-acquisition systems in order to successfully colonize host tissues [25]. Previous studies found that the level of intracellular iron is a signal for the expression of several virulence genes in *F. psychrophilum* [26], and it serves as a signal during biofilm development in *P. aeruginosa* [27] and *S. maltophilia* [28]. As the pathogen encounters nutrient deprivation condition during infection, its increased ability to sequester iron is able to enhance bacterial virulence and compromise the host defenses. Pathogenic bacteria encounter iron-limiting conditions in host tissues [28], and, similarly, aquatic bacteria living in biofilms are exposed to low free iron concentrations. It is very intriguing, from an epidemiological point of view, that calcium promoted iron acquisition mechanism in *F. columnare* which serve as enhanced virulence factors required for host colonization. The phenomena of upregulation of iron-acquisition in biofilm-bound cells has been alsoreported in *F. columnare* biofilm (Lange et al., 2018) although the expression of siderophore synthesis genes was only found in catfish mucus-stimulated biofilms. The authors have previously suggested that iron acquisition systems were important for biofilm formation (Lange et al., 2017). However, it needs to be noted that in both studies they used a culture medium that contained approximately 100 more CaCl_2_ than the medium used in our study, therefore it is impossible to discern the role of calcium in upregulating iron-acquisition genes from the role of biofilm. In phylogenetically distantly related bacteria, such as *P. aeruginosa* [29], and *Thermotoga maritima* [30] calcium has been linked to biofilm formation and upregulation of iron-acquisition genes thus suggesting that the effect of calcium in iron regulation is possibly a conserved mechanism across bacteria.

The type IX secretion system (T9SS) is an outmembrane protein secretion system that is widespread among members of the Phylum Bacteroidetes [31]. T9SS protein substrates have conserved C-terminal domains (CTDs) in common, which function as signaling during cargo transportation. Genetic manipulation of *Rgp*B (a surface-associated cysteine proteinase-coding gene) to remove the CTD resulted in the loss of posttranslational modification, surface attachment, and function of the enzyme [32]. In our study, Ca^2+^ supplementation enhanced the expression of the T9SS C-terminal target domain-containing proteins in calcium-supplemented samples. The cargo proteins that are transported by the T9SS is poorly characterized *in F. columnare* but based on their predictive function, we have identified several genes that encode for cargo proteins including a zinc-dependent metalloproteinase (WP_060383035.1). The upregulation of genes that code for T9SS cargo proteins P/Ca may partially explain the elevated virulence of *F. columnare* under high hardness (high Ca^2+^) conditions by facilitating extracellular transport of critical proteins involved in attachment and tissue colonization [6]. On the other hand, T9SS-related genes were downregulated in B/Ca when compared with P/Ca supporting the hypothesis that T9SS plays two roles, depending on the lifestyle of the bacteria (Lasica et al.,): means of movement as part of the gliding machinery in *Flavobacterium* spp. and secretion of virulence factors involved in attachment and colonization of pathogens. It is not surprising that genes involved in gliding motility are suppressed when cells are in living in biofilm.

## Conclusions

In summary, the transcriptome analysis revealed the specialized biofilm physiology including reduced metabolism and elevated oxidative stress tolerance. Main categories of DEGs include genes required for iron uptake, oxidative stress response, respiration pathway, and biosynthesis and secretion of extracellular products. Calcium supplementation promoted the T9SS protein secretion system and iron acquisition, both of which are considered critical virulence factors in the genus *Flavobacterium*. In addition, the transcriptional response (e.g. oxidative stress, iron acquisition) in biofilm seemed to be a conserved mechanism among even the distantly-related bacterial organism. Genetic manipulation of *F. columnare* has proven very difficult and, currently, there are not standard methods available to generate knockout mutants or gene deletions in this species. Future studies are needed to develop a robust set of genetic tools for the manipulation of this pathogen that will allow us to understand the role of some of the genes identified in this study in *F. columnare* pathogenesis.

## Methods

### Bacteria and culture conditions

Strain ALG-00-530 of *F. columnare* was used in this study. This strain was isolated from channel catfish in Alabama in 2000 [33], and it has been used in previous biofilm studies by our group [3]. Bacterium was stored at − 80 °C as glycerol stocks and routinely cultured on modified Shieh (MS) agar or broth with shaking (125 rpm) at 28 °C for 24–48 h [34]. Calcium concentration in the medium was adjusted to the desired value with CaCl_2_∙2H_2_O.

### Biofilm formation under different calcium concentrations

Biofilm was assessed in 250 ml Erlenmeyer flasks containing 100 ml of MS broth. Calcium concentration was adjusted to 0, 2.5, 4.5 and 6.5 mM with CaCl_2_∙2H_2_O. Approximately, 10^7^ CFU/ml of *F. columnare* ALG-00-530 were added to each flask from an overnight culture in MS (OD = 0.8). To allow for air exchange, the mouth of the flasks was covered with autoclaved cotton plugs. Cultures were incubated at 28 °C for 48 h under shaking.

### RNA extraction

Biofilm was induced in medium containing 4.5 mM [Ca^2+^] in flasks as described above. Planktonic cells (1 ml) from control (no calcium supplementation) and calcium-supplemented media were pelleted by centrifugation at 3000 rpm for 10 min at 4 °C. Biofilm formed at the interphase between air-liquid, around the edges of the flask. Using a sterilized spatula, approximately 0.05 g (wet weight) of biofilm was collected from the calcium-supplemented medium. No observable biofilm was produced in control flasks therefore it was not possible to study gene expression in biofilm in the absence of calcium. Planktonic cell pellets and biofilm samples were preserved in RNALater (Qiagen, Valencia, CA) at 4 °C and frozen at − 20 °C until processed. RNA was extracted using the TRIzol (Invitrogen, Waltham, MA) method with some modifications required to obtain sufficient RNA from biofilm samples. Briefly, samples were transferred from RNALater into a 2 ml tube (MP Biomedicals, Dayton, OH) containing 0.4 g of acid-washed 500 mm glass beads and 1 ml TRIzol solution. Samples were vortexed for 40 s and chilled down on ice for an additional 30 s. This process was repeated three times. Afterwards, 200 μl of chloroform was added to each sample and tubes were centrifuged at 12,000 g for 15 min at 4 °C. The supernatant was transferred to a new tube and washed again with equal volume of chloroform/isoamyl alcohol (24:1) solution. The aqueous phase was then carefully transferred to a new tube and mixed with equal volume of 100% ethanol. RNA was precipitated at − 20 °C for at least 30 min and washed twice in 75% ethanol before resuspended it into 60 μl of DEPC-water. RNA concentration and purity was spectrometrically determined using a NanoDrop 1000 (Thermo Scientific, Waltham, MA). RNA concentration and integrity were further assessed with a 2100 Bioanalyzer (Agilent Technologies, Santa Clara, CA) before proceeding to RNA sequencing. Three independent replicates were carried out per sample type.

### RNA sequencing

RNA-Seq library preparation was carried out by HudsonAlpha Genomic Service Lab (Huntsville, AL, USA). RNA integrity numbers (RIN) of the 9 samples were between 7.8–9.8 (average 9.2). Ribosomal RNA was removed from each sample using the RiboZero Gold Epidemiology rRNA Removal kit (Illumina, San Diego, CA). cDNA libraries were prepared through the NEBNext Ultra II synthesis module workflow (New England BioLabs, Ipswich, MA). The libraries were pooled and sequenced with 2 × 100 bp paired-end reads on an Illumina HiSeq2000 instrument.

### RNA-Seq data analysis

Adapter removal and quality trimming of FASTQ files was conducted using Trimmomatic [35] by the HudsonAlpha Genomic Service Lab. Data quality was assessed using FastQC version 0.10.1 (Babraham Bioinformatics). Reads were aligned to *F. columnare* strain 94–081 [36] protein-coding sequences (Accession number NZ_CP013992) using the Bowtie2 software [37]. The alignment statistics were obtained using Samtools [38]. Differentially expressed genes were identified using EdgeR [39]. Genes with an FDR-adjusted *p* value < 0.05, and fold change > 2 were identified as being differentially expressed. Functional annotation and gene ontology were also conducted using BLAST2Go PRO software [40]. Gene descriptions were annotated as in GeneBank of NCBI. When appropriate, gene annotations were updated with information from InterPro protein database by InterProScan 5 [41] for predicted functional domains.

### qPCR validation and statistical analysis

Nine genes that were up- or down-regulated by more than twofold were selected for qPCR validation of RNA-Seq data. Five genes were identified in the comparison P/Ca versus P and 4 genes were selected from the comparison B/Ca versus P/Ca. Genes were selected based on their functional annotations and were involved in iron acquisition, quorum sensing, and protein secretion. The primer pairs used in this study are listed in Additional file 2: Table S1. Total RNA (subsample from DNase-treated RNA samples that were used for sequencing) was reverse transcribed into cDNA with Applied Biosystems Reverse Transcription kit (Life Technologies Corporation, CA) according to the manufacture’s protocol. QPCR was carried out using Applied Biosystems 7500 Real-time PCR system with the following cycle conditions: 95 °C for 10 min, followed by 40 cycles of 95 °C for 15 s, and 60 °C for 60 s. Three biological replicates were used for RNA-seq treatments and subsequent qPCR validation. QPCR results were statistically analyzed using SAS software version 9.2 (SAS Institute, Cary, NC). The significant difference was set at *p* ≤ 0.05.

## Additional files

Additional file 1: Additional material includes three lists with differentially expressed genes (all data shown: RPM > 1, FDR < 0.05, and fold change > 2.0.). Supplemental Table 1. DEGs between *F. columnare* ALG-00-530 bacterial cells in biofilm compared to planktonic cells in 4.5 mM [Ca2+]. Supplemental Table 2. DEGs between *F. columnare* ALG-00-530 planktonic cells in planktonic cells in 4.5 mM [Ca2+] compared to planktonic cells in control medium. Supplemental Table 3. DEGs between *F. columnare* ALG-00-530 biofilm cells in 4.5 mM [Ca2+] medium compared to planktonic cells in control medium. (DOCX 95 kb)

Additional file 2: **Supplemental Table 4.** Primers used for qPCR. **Figure S1.** Library size for all samples. **Figure S2.** Heat-map of the top-50 DEGs expression profiles among the 9 samples after 48 h incubation. **Figure S3.** Gene ontology annotation of the up- (panel A) and down-regulated (panel B) genes between samples. (DOCX 992 kb)

## Data Availability

The datasets generated and analyzed during the current study are available from the corresponding author on reasonable request.

## Abbreviations

B: Biofilm cells
Ca: Cells in calcium-enriched medium
Ca2 +: Calcium
CM: Cytoplasmic membrane
CTD: C-terminal domain
DEGs: Differentially expressed genes
DNA: Deoxyribonucleic acid
OM: Outer membrane
P: Planktonic cells
PCA: Principal component analysis
QPCR: Quantitative polymerase chain reaction
RNA: Ribonucleic acid
ROS: Reactive oxygen species
USDA: United States Department of Agriculture
