# Supplementary material for: Transcriptome analysis of the fish pathogen Flavobacterium columnare in biofilm suggests calcium role in pathogenesis
Source: BMC Microbiol. 2019 Jul 4;19:151. doi: 10.1186/s12866-019-1533-4 (PMC6610971; doi:10.1186/s12866-019-1533-4)
Supplement: Supplementary file 1 — Additional material includes three lists with differentially expressed genes (all data shown: RPM > 1, FDR < 0.05, and fold change > 2.0.). Supplemental Table 1. DEGs between F. columnare ALG-00-530 bacterial cells in biofilm compared to planktonic cells in 4.5 mM [Ca2+]. Supplemental Table 2. DEGs between F. columnare ALG-00-530 planktonic cells in planktonic cells in 4.5 mM [Ca2+] compared to planktonic cells in control medium. Supplemental Table 3. DEGs between F. columnare ALG-00-530 biofilm cells in 4.5 mM [Ca2+] medium compared to planktonic cells in control medium. (DOCX 95 kb) [file 12866_2019_1533_MOESM1_ESM.docx]

**Supplementary Table 1**. DEGs between *F. columnare* ALG-00-530 bacterial cells in biofilm compared to planktonic cells in 4.5 mM [Ca2+]. Positive fold change values indicate higher expression in biofilm cells, while negative fold change values indicate higher expression in planktonic cells. All data shown: RPM >1, FDR < 0.05, and fold change > 2.0

| **Genes (coding sequences)** | **logFC** | **logCPM** | **PValue** | **FDR** | **Description** |
| --- | --- | --- | --- | --- | --- |
| WP_060382480.1 | -2.122 | 11.38171 | 7.45E-06 | 0.000135984 | cytochrome C oxidase subunit III |
| WP_060381433.1 | -2.106 | 7.843856 | 4.88E-06 | 9.58423E-05 | crystallin J1 |
| WP_060382479.1 | -2.033 | 10.9172 | 2.95E-06 | 6.28161E-05 | cytochrome c oxidase accessory protein CcoG |
| WP_060382475.1 | -1.998 | 7.842193 | 0.001355 | 0.009338842 | glycosyltransferase |
| WP_060381434.1 | -1.919 | 7.360791 | 6.55E-05 | 0.000872263 | hypothetical protein |
| WP_060381432.1 | -1.875 | 5.637942 | 6.87E-06 | 0.000127991 | hypothetical protein |
| WP_060381571.1 | -1.738 | 11.7915 | 0.0013 | 0.009091094 | quinol:cytochrome C oxidoreductase |
| WP_060381572.1 | -1.718 | 10.94184 | 0.001774 | 0.011591856 | hydrogenase |
| WP_060383513.1 | -1.717 | 2.727149 | 2.96E-08 | 1.23988E-06 | hypothetical protein |
| WP_060382481.1 | -1.714 | 8.692903 | 2.38E-05 | 0.000371322 | cytochrome C oxidase subunit IV |
| WP_060382478.1 | -1.714 | 9.798327 | 0.001935 | 0.012484625 | cytochrome C oxidase Cbb3 |
| WP_060383027.1 | -1.652 | 4.031734 | 0.004021 | 0.021299638 | hypothetical protein |
| WP_060381431.1 | -1.642 | 3.214371 | 5.50E-05 | 0.00075505 | hypothetical protein |
| WP_060382477.1 | -1.637 | 9.493366 | 1.28E-05 | 0.000216399 | hypothetical protein |
| WP_060382482.1 | -1.635 | 13.14644 | 5.80E-05 | 0.000784641 | cytochrome C oxidase Cbb3 |
| WP_060383512.1 | -1.606 | 2.557358 | 1.75E-06 | 3.95644E-05 | hypothetical protein |
| WP_060381573.1 | -1.569 | 9.590081 | 0.002927 | 0.01685227 | hypothetical protein |
| WP_060382476.1 | -1.546 | 11.27273 | 1.48E-05 | 0.000247361 | GTP-binding protein |
| WP_060383852.1 | -1.534 | 9.273206 | 5.60E-19 | 3.5199E-16 | branched-chain amino acid aminotransferase |
| WP_060381577.1 | -1.495 | 11.58669 | 0.003062 | 0.017312822 | cytochrome c oxidase subunit I |
| WP_060381419.1 | -1.446 | 10.52043 | 9.82E-05 | 0.001170532 | cytochrome c peroxidase |
| WP_060383027.1 | -1.410 | 3.981377 | 0.013934 | 0.053272219 | hypothetical protein |
| WP_060381575.1 | -1.401 | 10.82421 | 0.00445 | 0.022756906 | hypothetical protein |
| WP_060381570.1 | -1.392 | 10.16156 | 0.000367 | 0.003406465 | cytochrome C |
| WP_060381576.1 | -1.390 | 10.83432 | 0.005217 | 0.025837142 | cytochrome C oxidase subunit II |
| WP_060381574.1 | -1.373 | 9.451262 | 0.005553 | 0.026971442 | cytochrome C |
| WP_060382313.1 | -1.353 | 7.4134 | 2.71E-10 | 1.89626E-08 | anthranilate synthase |
| WP_060383400.1 | -1.304 | 2.093476 | 8.33E-05 | 0.001049196 | transposase |
| WP_060383120.1 | -1.288 | 8.671223 | 7.58E-05 | 0.000987788 | recombination protein RecR |
| WP_060381304.1 | -1.277 | 2.213413 | 3.72E-05 | 0.000544569 | transposase |
| WP_060381561.1 | -1.271 | 8.861832 | 0.000832 | 0.006497933 | NADH dehydrogenase |
| WP_060383583.1 | -1.232 | 6.282526 | 0.000375 | 0.00345867 | hypothetical protein |
| WP_060381631.1 | -1.191 | 7.472837 | 0.007194 | 0.032969489 | hypothetical protein |
| WP_060381560.1 | -1.185 | 8.793067 | 0.000767 | 0.006141917 | NADH oxidoreductase (quinone) subunit F |
| WP_060383582.1 | -1.180 | 6.350154 | 0.000269 | 0.002708993 | T9SS C-terminal target domain-containing protein |
| WP_060383834.1 | -1.171 | 5.675174 | 1.77E-08 | 8.11185E-07 | alpha/beta hydrolase |
| WP_060383838.1 | -1.169 | 6.217834 | 3.65E-06 | 7.52606E-05 | anthranilate synthase subunit II |
| WP_060383789.1 | -1.166 | -0.10001 | 0.008158 | 0.03613531 | hypothetical protein |
| WP_060383628.1 | -1.156 | 0.561693 | 0.00538 | 0.026438214 | transcriptional regulator |
| WP_060383402.1 | -1.131 | 9.053654 | 1.29E-06 | 3.06882E-05 | T9SS C-terminal target domain-containing protein |
| WP_060382408.1 | -1.121 | 8.942041 | 0.006089 | 0.029068003 | coproporphyrinogen III oxidase |
| WP_060382429.1 | -1.116 | 8.841991 | 4.84E-05 | 0.000680491 | MBL fold metallo-hydrolase |
| WP_060381558.1 | -1.074 | 8.570683 | 0.000893 | 0.00682904 | NADH dehydrogenase |
| WP_060381435.1 | -1.068 | 8.544385 | 8.72E-03 | 0.038025178 | phosphoribosylpyrophosphate synthetase |
| WP_060381566.1 | -1.067 | 8.766662 | 0.000156 | 0.001727037 | NADH-quinone oxidoreductase subunit L |
| WP_060381562.1 | -1.063 | 8.467717 | 0.001731 | 0.011429833 | NADH:ubiquinone oxidoreductase subunit H |
| WP_060383670.1 | -1.057 | 10.48085 | 0.009496 | 0.040631335 | succinate dehydrogenase |
| WP_060381563.1 | -1.055 | 7.100096 | 0.004 | 0.021232805 | DNA-binding protein |
| WP_060383119.1 | -1.034 | 8.639823 | 0.002774 | 0.016195425 | sugar transporter |
| WP_060382314.1 | -1.033 | 6.030786 | 2.67E-08 | 1.17725E-06 | anthranilate phosphoribosyltransferase |
| WP_014164161.1 | -1.003 | 7.489581 | 0.001318 | 0.009162692 | NADH-quinone oxidoreductase subunit I |
| WP_060382096.1 | 1.002 | 9.91266 | 8.34E-05 | 0.001049196 | band 7 protein |
| WP_060381444.1 | 1.008 | 10.16969 | 8.80E-05 | 0.001075239 | Nicotinamide mononucleotide adenylyltransferase |
| WP_060381969.1 | 1.024 | 6.949804 | 1.28E-13 | 2.93744E-11 | NAD(P)H-dependent oxidoreductase, Nitroreductase family |
| WP_060382110.1 | 1.028 | 4.646673 | 4.53E-05 | 0.000651384 | TetR family transcriptional regulator |
| WP_014164670.1 | 1.03174 | 7.219294 | 2.62E-11 | 2.35315E-09 | thiol reductase thioredoxin |
| WP_060382829.1 | 1.036 | 8.49837 | 2.75E-08 | 1.19494E-06 | hypothetical protein |
| WP_060383176.1 | 1.044 | 5.763719 | 2.44E-07 | 7.38941E-06 | hypothetical protein |
| WP_060381977.1 | 1.045 | 5.228536 | 1.33E-11 | 1.39432E-09 | protein-tyrosine-phosphatase |
| WP_060381978.1 | 1.047 | 4.374454 | 7.51E-09 | 3.85644E-07 | SAM-dependent methyltransferase |
| WP_060383765.1 | 1.050 | 6.64372 | 1.82E-06 | 4.05597E-05 | hypothetical protein |
| WP_060383508.1 | 1.056 | 7.135941 | 8.39E-09 | 4.21969E-07 | hypothetical protein |
| WP_060383025.1 | 1.057 | 4.208032 | 6.50E-06 | 0.000122917 | XRE-family like transcription factor |
| WP_060382470.1 | 1.063 | 5.952907 | 1.33E-07 | 4.59484E-06 | Ribosomal RNA large subunit methyltransferase H |
| WP_060381599.1 | 1.083 | 6.844685 | 8.21E-08 | 3.08147E-06 | hypothetical protein |
| WP_060381777.1 | 1.094 | 1.141965 | 0.000332 | 0.003137635 | hypothetical protein |
| WP_060383412.1 | 1.094 | 11.59059 | 3.91E-08 | 1.61314E-06 | signal peptide peptidase |
| WP_060382792.1 | 1.109 | 8.565248 | 8.86E-14 | 2.23023E-11 | peptidase M61 (glycyl aminopeptidase ) |
| WP_060382137.1 | 1.111 | 3.489552 | 0.000981 | 0.007298638 | RNA polymerase subunit sigma-70, transcriptional regulator |
| WP_060382994.1 | 1.115 | 8.530702 | 8.26E-10 | 5.06974E-08 | LemA family protein |
| WP_060381919.1 | 1.118 | 7.678662 | 5.54E-12 | 6.97559E-10 | O-succinylbenzoic acid--CoA ligase |
| WP_060381606.1 | 1.131 | 4.582837 | 2.58E-06 | 5.58605E-05 | hypothetical protein |
| WP_060382069.1 | 1.154 | 10.6691 | 9.92E-07 | 2.47072E-05 | hypothetical protein |
| WP_060381307.1 | 1.161 | 8.183417 | 0.000108 | 0.001269596 | hypothetical protein |
| WP_060381920.1 | 1.173 | 7.05944 | 7.95E-10 | 5.00307E-08 | abortive phage infection protein. |
| WP_060382943.1 | 1.178 | 4.225066 | 9.15E-09 | 4.34352E-07 | hypothetical protein |
| WP_014165796.1 | 1.189 | 4.357067 | 1.37E-06 | 3.18357E-05 | hypothetical protein |
| WP_060382136.1 | 1.210 | 4.140716 | 1.98E-07 | 6.14642E-06 | hypothetical protein |
| WP_060381633.1 | 1.236 | 8.897917 | 2.84E-13 | 5.48987E-11 | hypothetical protein |
| WP_060381836.1 | 1.245 | 3.540121 | 1.82E-06 | 4.05597E-05 | UDP-N-acetylmuramate--alanine ligase |
| WP_060381311.1 | 1.255 | 8.660461 | 6.16E-09 | 3.36707E-07 | histidine kinase.phosphotransfer |
| WP_060381932.1 | 1.260 | 8.707618 | 2.65E-10 | 1.89626E-08 | hypothetical protein,BlaR1 peptidase M56 |
| WP_060381976.1 | 1.263 | 6.765991 | 7.81E-17 | 2.80603E-14 | hypothetical protein |
| WP_060382995.1 | 1.267 | 1.310361 | 6.79E-05 | 0.000894451 | hypothetical protein |
| WP_060381532.1 | 1.271 | 7.185773 | 3.74E-10 | 2.54308E-08 | enoyl-CoA hydratase |
| WP_060382395.1 | 1.283 | 4.718922 | 4.95E-06 | 9.66089E-05 | hypothetical protein |
| WP_060382125.1 | 1.293 | 2.253881 | 1.96E-05 | 0.000318841 | hypothetical protein |
| WP_060382782.1 | 1.297 | 5.007814 | 6.16E-06 | 0.000117556 | hypothetical protein |
| WP_060382836.1 | 1.303 | 6.174188 | 1.96E-16 | 6.15065E-14 | hypothetical protein |
| WP_060383199.1 | 1.307 | 4.355146 | 8.46E-12 | 9.26542E-10 | hypothetical protein, ribosome inactivating protein |
| WP_060381283.1 | 1.313 | 6.292429 | 1.80E-12 | 2.83439E-10 | single-stranded DNA-binding protein |
| WP_060381931.1 | 1.314 | 4.356933 | 5.39E-08 | 2.11768E-06 | transcriptional regulator |
| WP_060382837.1 | 1.319 | 6.175612 | 1.64E-17 | 8.228E-15 | cell division protein ZapA |
| WP_060382135.1 | 1.347 | 5.208551 | 5.48E-11 | 4.75195E-09 | hypothetical protein |
| WP_060381968.1 | 1.358 | 5.890936 | 1.79E-12 | 2.83439E-10 | TetR family transcriptional regulator |
| WP_060382698.1 | 1.385 | 9.206716 | 1.85E-07 | 5.96342E-06 | macrolide ABC transporter ATP-binding protein |
| WP_060381979.1 | 1.388 | 3.807785 | 2.03E-10 | 1.50705E-08 | hypothetical protein |
| WP_060382095.1 | 1.413 | 5.622364 | 7.87E-16 | 2.20128E-13 | MarR family transcriptional regulator. |
| WP_060382768.1 | 1.413 | 3.623763 | 1.89E-07 | 6.01795E-06 | Hypothetical protein |
| WP_060382235.1 | 1.414 | 4.935591 | 7.36E-13 | 1.32291E-10 | Cytochrome c peroxidase |
| WP_060381835.1 | 1.427 | 3.316255 | 1.44E-11 | 1.44471E-09 | Hypothetical protein |
| WP_060382622.1 | 1.456 | 10.19297 | 1.99E-11 | 1.85347E-09 | TonB-dependent receptor |
| WP_060383353.1 | 1.497 | 9.133181 | 2.90E-12 | 4.04796E-10 | ABC transporter permease |
| WP_060381975.1 | 1.503 | 9.415081 | 1.98E-17 | 8.28874E-15 | alkyl hydroperoxide reductase |
| WP_060382658.1 | 1.540 | 9.216781 | 4.40E-12 | 5.83312E-10 | von willebrand factore type protein |
| WP_060381980.1 | 1.544 | 2.901708 | 3.11E-05 | 0.000466474 | ArsR family transcriptional regulator. |
| WP_060382775.1 | 1.566 | 10.66737 | 2.59E-07 | 7.76803E-06 | TonB-dependent receptor plug domain |
| WP_060382614.1 | 1.706 | 3.763124 | 1.41E-13 | 2.9658E-11 | AraC family transcriptional regulator |
| WP_060381967.1 | 1.715 | 6.314766 | 1.25E-22 | 1.0517E-19 | Glycoside hydrolase, 1,4-polygalactosaminidase |
| WP_060382447.1 | 1.755 | 8.059357 | 2.34E-27 | 5.89051E-24 | Hypothetical protein(contain LETM1-like protein) |
| WP_060382448.1 | 2.076 | 7.458086 | 6.61E-26 | 8.31318E-23 | DNA starvation protect protein |
| WP_060381974.1 | 2.159 | 4.697825 | 7.27E-12 | 8.71409E-10 | Catalase |

**Supplementary Table 2**. DEGs between *F. columnare* ALG-00-530 planktonic cells in 4.5 mM [Ca2+] compared to planktonic cells in control medium. Positive fold change values indicate higher expression in planktonic cells in 4.5 mM [Ca2+], while negative fold change values indicate higher expression in planktonic cells in control medium.

| **Genes (coding sequences)** | **logFC** | **logCPM** | **P value** | **FDR** | **Description** |
| --- | --- | --- | --- | --- | --- |
| WP_060383188.1 | 8.320 | 9.760374 | 8.48E-46 | 2.13395E-43 | Membrane protein |
| WP_060383187.1 | 8.202 | 10.87413 | 1.96E-43 | 4.10694E-41 | siderophore alcaligin biosynthesis protein |
| WP_060383189.1 | 7.991 | 10.96539 | 6.62E-56 | 3.33363E-53 | N-acetyltransferase |
| WP_060383186.1 | 7.974 | 10.58058 | 7.84E-58 | 6.57887E-55 | aspartate aminotransferase family protein |
| WP_060382679.1 | 7.607 | 11.59612 | 3.51E-45 | 8.03004E-43 | T9SS C-terminal target domain-containing protein |
| WP_060382680.1 | 7.370 | 10.54294 | 2.19E-66 | 5.52094E-63 | hypothetical protein |
| WP_060383888.1 | 7.207 | 9.176353 | 5.09E-64 | 6.40737E-61 | siderophore biosynthesis protein |
| WP_060382681.1 | 6.364 | 11.17095 | 7.61E-54 | 2.73526E-51 | hypothetical protein |
| WP_060383861.1 | 6.030 | 10.03701 | 3.60E-42 | 6.96342E-40 | TonB-dependent receptor |
| WP_060383182.1 | 6.008 | 11.59184 | 1.40E-51 | 4.41246E-49 | TonB-dependent receptor |
| WP_060383190.1 | 5.642 | 8.853831 | 6.29E-47 | 1.75954E-44 | lucA/lucC family siderophore biosynthesis protein |
| WP_060381363.1 | 5.616 | 9.930441 | 1.49E-56 | 9.35274E-54 | hypothetical protein |
| WP_060381364.1 | 5.483 | 10.49137 | 1.74E-55 | 7.28177E-53 | hypothetical protein |
| WP_060383101.1 | 5.179 | 11.78905 | 2.38E-35 | 3.73784E-33 | TonB-dependent receptor (siderophore transport) |
| WP_060383184.1 | 4.585 | 6.494663 | 2.94E-37 | 5.28392E-35 | hypothetical protein |
| WP_060383183.1 | 4.453 | 8.909717 | 1.45E-35 | 2.43226E-33 | peptidase M4 |
| WP_060383191.1 | 3.944 | 8.102602 | 1.76E-30 | 2.45874E-28 | hypothetical protein |
| WP_060383185.1 | 3.943 | 6.20772 | 1.88E-31 | 2.7841E-29 | LuxR family transcriptional regulator |
| WP_060382675.1 | 3.697 | 9.826139 | 1.07E-25 | 1.17568E-23 | PadR family transcriptional regulator |
| WP_060382006.1 | 3.656 | 7.052608 | 4.95E-19 | 3.88823E-17 | MarR family transcriptional regulator |
| WP_060382676.1 | 3.630 | 12.02067 | 2.17E-26 | 2.59948E-24 | hypothetical protein |
| WP_060381858.1 | 3.517 | 6.351945 | 4.31E-24 | 4.16613E-22 | hypothetical protein |
| WP_060383100.1 | 3.484 | 8.472096 | 4.26E-26 | 4.86861E-24 | hypothetical protein |
| WP_060382677.1 | 3.451 | 9.86124 | 2.02E-26 | 2.54365E-24 | hypothetical protein |
| WP_060382674.1 | 3.440 | 9.665114 | 5.49E-22 | 4.93057E-20 | hypothetical protein |
| WP_060382672.1 | 3.407 | 6.791994 | 3.84E-27 | 5.08558E-25 | thioesterase |
| WP_060382467.1 | 3.284 | 11.30332 | 6.93E-23 | 6.46079E-21 | DUF4856 domain-containing protein |
| WP_060382673.1 | 3.098 | 9.88809 | 1.57E-15 | 9.61463E-14 | hypothetical protein |
| WP_060382775.1 | 2.971 | 10.66737 | 1.76E-20 | 1.53113E-18 | TonB-dependent receptor |
| WP_060381365.1 | 2.954 | 9.564651 | 1.82E-24 | 1.83593E-22 | ArsR family transcriptional regulator |
| WP_060382096.1 | 2.816 | 9.91266 | 2.03E-25 | 2.13007E-23 | band 7 protein |
| WP_060382588.1 | 2.792 | 9.476391 | 2.11E-20 | 1.77107E-18 | phosphate transport regulator |
| WP_060382504.1 | 2.784 | 5.862975 | 3.13E-17 | 2.07236E-15 | hypothetical protein |
| WP_060383099.1 | 2.763 | 6.906051 | 3.10E-20 | 2.51538E-18 | flagellin biosynthesis protein FlgD |
| WP_060383004.1 | 2.616 | 3.757444 | 1.03E-10 | 3.32918E-09 | hypothetical protein |
| WP_060381538.1 | 2.596 | 11.62834 | 1.51E-18 | 1.08364E-16 | hypothetical protein (cholesterol binding) |
| WP_060383657.1 | 2.446 | 5.62707 | 6.42E-15 | 3.75496E-13 | ion transporter |
| WP_060382466.1 | 2.438 | 8.818583 | 6.77E-19 | 5.01185E-17 | hypothetical protein |
| WP_060382034.1 | 2.415 | 8.161425 | 2.00E-18 | 1.39438E-16 | hypothetical protein(integral component of membrane) |
| WP_060381366.1 | 2.290 | 8.277305 | 2.06E-15 | 1.237E-13 | two-component sensor histidine kinase |
| WP_060382698.1 | 2.283 | 9.206716 | 7.58E-17 | 4.89036E-15 | macrolide ABC transporter ATP-binding protein |
| WP_060382550.1 | 2.208 | 8.998759 | 5.57E-19 | 4.24797E-17 | glycerol kinase |
| WP_060381668.1 | 2.190 | 7.619535 | 1.11E-12 | 4.71305E-11 | 2-nitropropane dioxygenase |
| WP_060383037.1 | 2.174 | 11.24629 | 1.89E-12 | 7.65027E-11 | hypothetical protein |
| WP_060382587.1 | 2.139 | 10.5364 | 4.25E-14 | 2.32648E-12 | phosphate transporter |
| WP_060382005.1 | 2.131 | 10.94988 | 1.57E-11 | 5.55611E-10 | 3-hydroxyacyl-CoA dehydrogenase |
| WP_060382870.1 | 2.063 | 10.4933 | 1.24E-17 | 8.43503E-16 | esterase |
| WP_060381307.1 | 2.032 | 8.183417 | 4.37E-11 | 1.48429E-09 | hypothetical protein |
| WP_060382465.1 | 2.019 | 7.017035 | 1.28E-12 | 5.35783E-11 | deoxyribonuclease HsdR |
| WP_060383098.1 | 1.920 | 7.473981 | 3.53E-12 | 1.38749E-10 | hypothetical protein |
| WP_060383327.1 | 1.901 | 10.64187 | 6.23E-12 | 2.3745E-10 | electron transfer flavoprotein subunit alpha |
| WP_060382549.1 | 1.817 | 7.602577 | 3.86E-16 | 2.42706E-14 | FAD-dependent oxidoreductase (glycerol related) |
| WP_060383583.1 | 1.811 | 6.282526 | 2.72E-07 | 5.65284E-06 | hypothetical protein |
| WP_060383369.1 | 1.777 | 8.702484 | 1.45E-09 | 4.13406E-08 | thioredoxin |
| WP_060383795.1 | 1.747 | 8.877857 | 3.51E-14 | 1.96336E-12 | RNA-splicing ligase RtcB |
| WP_060383674.1 | 1.714 | 12.90898 | 2.17E-14 | 1.24251E-12 | X-Pro aminopeptidase |
| WP_014164868.1 | 1.701 | 11.06844 | 8.26E-12 | 3.05734E-10 | RNA polymerase sigma factor RpoD |
| WP_060383854.1 | 1.698 | 6.147511 | 3.97E-13 | 1.91922E-11 | aquaporin (water channel protein) |
| WP_060382871.1 | 1.669 | 9.506231 | 7.24E-14 | 3.79483E-12 | acetyltransferase |
| WP_060383036.1 | 1.658 | 10.21155 | 8.81E-08 | 1.97834E-06 | cyclase(arylformamidase activity) |
| WP_060383035.1 | 1.652 | 6.557008 | 1.90E-11 | 6.63111E-10 | T9SS C-terminal target domain-containing protein |
| WP_060383786.1 | 1.644 | 11.71884 | 7.58E-13 | 3.33948E-11 | endothelin-converting protein |
| WP_060383326.1 | 1.639 | 10.73342 | 3.09E-09 | 8.43985E-08 | electron transfer flavoprotein subunit alpha |
| WP_060383401.1 | 1.613 | 9.349299 | 6.22E-13 | 2.89982E-11 | hypothetical protein |
| WP_060383376.1 | 1.595 | 13.38785 | 3.10E-11 | 1.06765E-09 | hypothetical protein |
| WP_060383582.1 | 1.576 | 6.350154 | 1.48E-06 | 2.79601E-05 | T9SS C-terminal target domain-containing protein |
| WP_060383353.1 | 1.564 | 9.133181 | 3.82E-13 | 1.88387E-11 | ABC transporter permease |
| WP_060382069.1 | 1.540 | 10.6691 | 1.03E-10 | 3.32918E-09 | hypothetical protein |
| WP_060383656.1 | 1.532 | 7.805838 | 9.73E-11 | 3.21976E-09 | ferrous iron transport protein B |
| WP_060382464.1 | 1.528 | 7.73979 | 5.41E-12 | 2.09235E-10 | TonB-dependent receptor |
| WP_060381901.1 | 1.521 | 7.581453 | 7.55E-12 | 2.83625E-10 | hypothetical protein |
| WP_060381835.1 | 1.520 | 3.316255 | 5.93E-10 | 1.79762E-08 | hypothetical protein |
| WP_060381532.1 | 1.519 | 7.185773 | 1.74E-13 | 8.91429E-12 | enoyl-CoA hydratase |
| WP_060382699.1 | 1.511 | 12.33655 | 1.69E-09 | 4.72496E-08 | peptidase M16 |
| WP_060381689.1 | 1.474 | 10.37171 | 1.35E-07 | 2.93357E-06 | flagellar motor protein MotA |
| WP_060383434.1 | 1.460 | 9.679931 | 7.64E-10 | 2.28724E-08 | outmembrane efflux protein |
| WP_060381632.1 | 1.426 | 8.668103 | 9.65E-10 | 2.82206E-08 | hypothetical protein |
| WP_060383675.1 | 1.421 | 9.746996 | 1.12E-11 | 4.06766E-10 | hypothetical protein |
| WP_060381383.1 | 1.420 | 8.912427 | 3.37E-13 | 1.69579E-11 | hypothetical protein |
| WP_060381306.1 | 1.394 | 8.608299 | 7.70E-13 | 3.33948E-11 | sugar isomerase |
| WP_060383550.1 | 1.386 | 3.830284 | 2.25E-05 | 0.000303011 | 1-acyl-sn-glycerol-3-phosphate acyltransferase |
| WP_060382995.1 | 1.373 | 1.310361 | 0.000598 | 0.004543301 | hypothetical protein |
| WP_060382817.1 | 1.370 | 7.837991 | 1.78E-06 | 3.29639E-05 | hypothetical protein |
| WP_060382624.1 | 1.367 | 8.6717 | 3.06E-10 | 9.73656E-09 | hypothetical protein |
| WP_060382678.1 | 1.339 | 7.949126 | 8.97E-10 | 2.65544E-08 | hypothetical protein |
| WP_060382004.1 | 1.325 | 9.591382 | 7.91E-05 | 0.000888648 | acetyl-CoA acetyltransferase |
| WP_060382994.1 | 1.321 | 8.530702 | 5.77E-13 | 2.73746E-11 | LemA family protein |
| WP_060382503.1 | 1.310 | 2.607642 | 4.47E-07 | 9.136E-06 | hypothetical protein |
| WP_060382314.1 | 1.308 | 6.030786 | 2.84E-12 | 1.13509E-10 | anthranilate phosphoribosyltransferase |
| WP_060382366.1 | 1.279 | 6.787778 | 3.50E-09 | 9.47371E-08 | hypothetical protein |
| WP_060381383.1 | 1.266 | 10.28724 | 3.37E-10 | 1.06122E-08 | hypothetical protein |
| WP_060382586.1 | 1.258 | 6.063765 | 4.44E-10 | 1.37906E-08 | hypothetical protein |
| WP_060382313.1 | 1.241 | 7.4134 | 6.38E-09 | 1.63814E-07 | anthranilate synthase |
| WP_060382997.1 | 1.237 | 6.389905 | 4.03E-09 | 1.0781E-07 | peptidase M23 |
| WP_060382993.1 | 1.234 | 5.870052 | 6.74E-13 | 3.0835E-11 | hypothetical protein |
| WP_060382315.1 | 1.227 | 5.160034 | 1.22E-08 | 3.01053E-07 | indole-3-glycerol phosphate synthase |
| WP_060383661.1 | 1.215 | 6.832323 | 4.99E-08 | 1.15215E-06 | patatin (lipase or storing function) |
| WP_060383370.1 | 1.214 | 9.068602 | 6.40E-06 | 0.000105881 | thioredoxin |
| WP_060382704.1 | 1.214 | 3.778093 | 9.23E-07 | 1.7857E-05 | hypothetical protein |
| WP_060381785.1 | 1.213 | 3.042683 | 1.23E-05 | 0.000177248 | hypothetical protein |
| WP_060383433.1 | 1.208 | 10.60813 | 6.78E-09 | 1.72317E-07 | efflux transporter periplasmic adaptor subunit |
| WP_060381373.1 | 1.189 | 7.109908 | 4.74E-10 | 1.4553E-08 | NifU family protein (iron-sulfur cluster binding) |
| WP_060381615.1 | 1.178 | 8.624158 | 5.85E-09 | 1.51711E-07 | hypothetical protein |
| WP_060381372.1 | 1.169 | 9.168523 | 8.87E-09 | 2.2328E-07 | chromosome partitioning protein(ATPase activity) |
| WP_060381757.1 | 1.163 | 8.590069 | 1.31E-08 | 3.20997E-07 | hypothetical protein |
| WP_060381679.1 | 1.155 | 2.476874 | 0.001071 | 0.007127348 | hypothetical protein |
| WP_060381392.1 | 1.149 | 3.139791 | 1.02E-05 | 0.000154959 | hypothetical protein |
| WP_060382957.1 | 1.141 | 4.485988 | 1.11E-05 | 0.000166149 | hypothetical protein |
| WP_060383668.1 | 1.135 | 7.869841 | 5.64E-09 | 1.47895E-07 | thioredoxin |
| WP_060383838.1 | 1.133 | 6.217834 | 7.05E-06 | 0.000115978 | anthranilate synthase subunit II |
| WP_060383676.1 | 1.133 | 8.192526 | 7.02E-13 | 3.15472E-11 | alpha/beta hydrolase |
| WP_060383689.1 | 1.120 | 6.136414 | 1.15E-09 | 3.31403E-08 | transcriptional regulator |
| WP_060382941.1 | 1.109 | 7.16821 | 1.52E-05 | 0.000214734 | hypothetical protein |
| WP_060382066.1 | 1.102 | 3.958931 | 2.62E-09 | 7.23208E-08 | hypothetical protein |
| WP_060381305.1 | 1.101 | 8.374591 | 8.77E-11 | 2.94082E-09 | glycosyl transferase family 1 |
| WP_060383910.1 | 1.095 | 5.996754 | 9.83E-08 | 2.1702E-06 | hypothetical protein |
| WP_060383628.1 | 1.095 | 0.740619 | 0.011824 | 0.042729572 | transcriptional regulator |
| WP_060383810.1 | 1.095 | 1.835 | 0.000852 | 0.005980807 | hypothetical protein |
| WP_060383338.1 | 1.094 | 5.146889 | 8.37E-06 | 0.000132398 | hypothetical protein |
| WP_060382840.1 | 1.092 | 8.299183 | 2.32E-05 | 0.000309031 | hypothetical protein |
| WP_060382136.1 | 1.090 | 4.140716 | 7.75E-06 | 0.000125038 | hypothetical protein |
| WP_060382622.1 | 1.08948 | 10.19297 | 4.28E-07 | 8.83011E-06 | TonB-dependent receptor |
| WP_060381443.1 | 1.075 | 12.23509 | 1.64E-06 | 3.06135E-05 | NADH-dependent alcohol dehydrogenase |
| WP_060382067.1 | 1.074 | 4.920698 | 9.25E-09 | 2.3055E-07 | hypothetical protein |
| WP_060383124.1 | 1.053 | 8.360609 | 1.59E-09 | 4.50235E-08 | antibiotic resistance protein MarC |
| WP_060381635.1 | 1.039 | 1.851533 | 0.002155 | 0.011711574 | hypothetical protein |
| WP_060383504.1 | 1.030 | 4.785884 | 1.17E-05 | 0.000170416 | methylmalonyl-CoA epimerase |
| WP_060383579.1 | 1.029 | 5.780629 | 2.40E-08 | 5.68623E-07 | hypothetical protein |
| WP_060383222.1 | 1.024 | 6.678646 | 1.51E-06 | 2.83261E-05 | 3-oxoacyl-ACP synthase |
| WP_060383545.1 | 1.018461 | 11.31238 | 1.18E-05 | 0.00017183 | TonB-dependent receptor |
| WP_060383420.1 | 1.016 | 6.173865 | 7.87E-06 | 0.000126057 | hypothetical protein |
| WP_060383337.1 | 1.015 | 4.600515 | 1.15E-05 | 0.000169084 | hypothetical protein |
| WP_060381611.1 | 1.009 | 3.978543 | 5.40E-07 | 1.07902E-05 | hypothetical protein |
| WP_060382985.1 | 1.009 | 0.714198 | 0.005067 | 0.022846938 | hypothetical protein |
| WP_060383589.1 | 1.004 | 9.676746 | 2.31E-07 | 4.93155E-06 | glutamyl-tRNA reductase |
| WP_060383259.1 | -1.010 | 13.11652 | 0.003022 | 0.01520807 | gliding motility protein GldN |
| WP_060381562.1 | -1.013 | 8.467717 | 0.002767 | 0.014210105 | NADH:ubiquinone oxidoreductase subunit H |
| WP_060381556.1 | -1.027 | 7.947913 | 0.000264 | 0.002425266 | NADH dehydrogenase |
| WP_060381594.1 | -1.033 | 7.30669 | 0.000347 | 0.002989039 | hypothetical protein |
| WP_060381797.1 | -1.055 | 6.051312 | 0.003222 | 0.015987033 | sulfate transporter |
| WP_060382476.1 | -1.064 | 11.27273 | 0.002549 | 0.013362758 | GTP-binding protein |
| WP_060383671.1 | -1.096 | 8.462906 | 2.60E-05 | 0.00034207 | succinate dehydrogenase |
| WP_060381560.1 | -1.117 | 8.793067 | 0.001494 | 0.009194884 | NADH oxidoreductase (quinone) subunit F |
| WP_060381555.1 | -1.117 | 7.116917 | 0.000731 | 0.005316854 | NADH-quinone oxidoreductase subunit A |
| WP_060382819.1 | -1.129 | 7.985046 | 1.33E-11 | 4.78709E-10 | carbamoyl phosphate synthase large subunit |
| WP_060383669.1 | -1.129 | 9.174589 | 0.002497 | 0.013145165 | fumarate reductase |
| WP_060381557.1 | -1.134 | 7.07777 | 0.000133 | 0.001407973 | NADH dehydrogenase |
| WP_060381795.1 | -1.156 | 6.808492 | 0.002248 | 0.012080798 | hypothetical protein |
| WP_060382918.1 | -1.168 | 4.182993 | 0.001415 | 0.008789671 | hypothetical protein |
| WP_060381558.1 | -1.192 | 8.570683 | 0.000233 | 0.002198883 | NADH dehydrogenase |
| WP_060381577.1 | -1.200 | 11.58669 | 0.016609 | 0.054538 | cytochrome c oxidase subunit I |
| WP_060382916.1 | -1.202 | 4.697264 | 0.000728 | 0.005307985 | hypothetical protein |
| WP_060382251.1 | -1.208 | 6.135555 | 9.44E-08 | 2.10142E-06 | lipoyl synthase |
| WP_060382917.1 | -1.220 | 5.311126 | 4.78E-05 | 0.000575157 | TonB-dependent receptor |
| WP_060382250.1 | -1.225 | 7.310715 | 0.000359 | 0.003083122 | hybrid sensor histidine kinase/response regulator |
| WP_060382477.1 | -1.241 | 9.493366 | 0.000824 | 0.005843194 | hypothetical protein |
| WP_060382302.1 | -1.244 | 2.916828 | 0.000745 | 0.005386529 | hypothetical protein |
| WP_060381563.1 | -1.263 | 7.100096 | 0.000595 | 0.004538488 | DNA-binding protein |
| WP_060382498.1 | -1.279 | 9.129416 | 3.80E-06 | 6.54039E-05 | zinc metalloprotease |
| WP_060381576.1 | -1.288 | 10.83432 | 0.009439 | 0.035777542 | cytochrome C oxidase subunit II |
| WP_060381631.1 | -1.290 | 7.472837 | 0.003652 | 0.017738317 | hypothetical protein |
| WP_060383670.1 | -1.299 | 10.48085 | 0.001518 | 0.009268724 | succinate dehydrogenase |
| WP_060381559.1 | -1.306 | 7.443497 | 0.000185 | 0.00180542 | NADH dehydrogenase |
| WP_060381574.1 | -1.307 | 9.451262 | 0.008156 | 0.032012578 | cytochrome C |
| WP_060382919.1 | -1.314 | 7.588117 | 0.001398 | 0.008725461 | cytochrome-c peroxidase |
| WP_060381575.1 | -1.316 | 10.82421 | 0.007404 | 0.029709909 | quinol:cytochrome C oxidoreductase |
| WP_060381573.1 | -1.327 | 9.590081 | 0.011321 | 0.041341716 | hypothetical protein |
| WP_060383835.1 | -1.347 | 7.324971 | 0.000171 | 0.001712941 | type I glyceraldehyde-3-phosphate dehydrogena |
| WP_060381796.1 | -1.400 | 5.819922 | 0.001004 | 0.006735549 | NADH dehydrogenase |
| WP_060381571.1 | -1.415 | 11.7915 | 0.008237 | 0.032279367 | quinol:cytochrome C oxidoreductase |
| WP_060382920.1 | -1.451 | 9.592328 | 0.01141 | 0.041595661 | hypothetical protein |
| WP_060382922.1 | -1.472 | 9.165178 | 0.005124 | 0.023021482 | hypothetical protein |
| WP_060381570.1 | -1.482 | 10.16156 | 0.000154 | 0.001575995 | cytochrome C |
| WP_060383214.1 | -1.593 | 4.482674 | 0.002004 | 0.011130643 | hypothetical protein |
| WP_060382921.1 | -1.596 | 9.925068 | 1.22E-02 | 0.043791953 | TonB-dependent receptor |

**Supplementary Table 3**. DEGs between *F. columnare* ALG-00-530 biofilm cells in 4.5 mM [Ca2+] medium compared to planktonic cells in control medium. Positive fold change values indicate higher expression in the biofilm cells in 4.5 mM [Ca2+] medium, while negative fold change values indicate higher expression in planktonic cells in control medium.

| **Genes (coding sequences)** | **logFC** | **logCPM** | **PValue** | **FDR** | **Description** |
| --- | --- | --- | --- | --- | --- |
| WP_060382475.1 | -3.175104 | 7.842193 | 1.14E-06 | 5.81327E-06 | glycosyltransferase |
| WP_060381571.1 | -3.15291 | 11.7915 | 3.22E-08 | 2.28644E-07 | quinol:cytochrome C oxidoreductase |
| WP_060382480.1 | -3.085468 | 11.38171 | 3.59E-10 | 3.81122E-09 | Cytochrome c |
| WP_060381572.1 | -2.986891 | 10.94184 | 2.18E-07 | 1.27184E-06 | hydrogase gene |
| WP_060382479.1 | -2.940048 | 10.9172 | 6.73E-11 | 7.98497E-10 | cytochrome c oxidase accessory protein CcoG |
| WP_060381573.1 | -2.896434 | 9.590081 | 1.63E-07 | 9.78896E-07 | hypothetical protein |
| WP_060382477.1 | -2.878 | 9.493366 | 2.20E-13 | 3.73907E-12 | hypothetical protein |
| WP_060381570.1 | -2.87406 | 10.16156 | 2.82E-12 | 4.19183E-11 | cytochrome C |
| WP_060383214.1 | -2.799708 | 4.482674 | 2.16E-07 | 1.26167E-06 | hypothetical protein |
| WP_060381575.1 | -2.716445 | 10.82421 | 1.33E-07 | 8.07399E-07 | hypothetical protein |
| WP_060381577.1 | -2.695092 | 11.58669 | 3.01E-07 | 1.70431E-06 | cytochrome c oxidase subunit I |
| WP_060381574.1 | -2.680523 | 9.451262 | 2.20E-07 | 1.2797E-06 | cytochrome C |
| WP_060381576.1 | -2.677406 | 10.83432 | 2.55E-07 | 1.46717E-06 | cytochrome C oxidase subunit II |
| WP_060382476.1 | -2.610057 | 11.27273 | 1.79E-12 | 2.78047E-11 | GTP-binding protein |
| WP_060382481.1 | -2.565137 | 8.692903 | 8.25E-10 | 8.11119E-09 | cytochrome C oxidase subunit IV |
| WP_060381631.1 | -2.481027 | 7.472837 | 7.34E-08 | 4.79513E-07 | hypothetical protein |
| WP_060382478.1 | -2.42863 | 9.798327 | 1.82E-05 | 7.09996E-05 | cytochrome C oxidase Cbb3 |
| WP_060382482.1 | -2.423004 | 13.14644 | 6.54E-09 | 5.43426E-08 | cytochrome C oxidase Cbb3 |
| WP_060382484.1 | -2.384431 | 8.426883 | 5.47E-05 | 0.000191925 | ATPase |
| WP_060381796.1 | -2.379264 | 5.819922 | 5.98E-08 | 3.99356E-07 | NADH dehydrogenase |
| WP_060381419.1 | -2.377757 | 10.52043 | 5.15E-10 | 5.29301E-09 | cytochrome-c peroxidase |
| WP_060383670.1 | -2.355933 | 10.48085 | 2.64E-08 | 1.92841E-07 | succinate dehydrogenase |
| WP_060383215.1 | -2.32334 | 3.321442 | 0.000109 | 0.000354327 | hypothetical protein |
| WP_060381563.1 | -2.318401 | 7.100096 | 1.04E-09 | 9.96129E-09 | DNA-binding protein |
| WP_060381560.1 | -2.301909 | 8.793067 | 2.61E-10 | 2.85762E-09 | NADH oxidoreductase (quinone) subunit F |
| WP_060381434.1 | -2.27928 | 7.360791 | 2.82E-06 | 1.31753E-05 | hypothetical protein |
| WP_060381558.1 | -2.266316 | 8.570683 | 1.26E-11 | 1.63701E-10 | NADH dehydrogenase |
| WP_060381433.1 | -2.265357 | 7.843856 | 1.01E-06 | 5.22398E-06 | crystallin J1 |
| WP_060381561.1 | -2.244439 | 8.861832 | 1.01E-08 | 8.16114E-08 | NADH dehydrogenase |
| WP_060381559.1 | -2.175028 | 7.443497 | 1.31E-09 | 1.24047E-08 | NADH dehydrogenase |
| WP_060382921.1 | -2.148233 | 9.925068 | 0.00092 | 0.002373301 | TonB-dependent receptor |
| WP_060381795.1 | -2.089765 | 6.808492 | 7.54E-08 | 4.88358E-07 | hypothetical protein |
| WP_060381562.1 | -2.076299 | 8.467717 | 2.62E-09 | 2.32134E-08 | NADH:ubiquinone oxidoreductase subunit H |
| WP_060383669.1 | -2.036009 | 9.174589 | 1.08E-07 | 6.76676E-07 | fumarate reductase |
| WP_060381566.1 | -2.035379 | 8.766662 | 2.07E-12 | 3.15904E-11 | NADH-quinone oxidoreductase subunit L |
| WP_060381431.1 | -1.964525 | 3.214371 | 1.58E-06 | 7.79944E-06 | hypothetical protein |
| WP_060381555.1 | -1.952004 | 7.116917 | 7.96E-09 | 6.56321E-08 | NADH-quinone oxidoreductase subunit A |
| WP_014164161.1 | -1.934572 | 7.489581 | 1.42E-09 | 1.3295E-08 | NADH-quinone oxidoreductase subunit I |
| WP_060381557.1 | -1.929302 | 7.07777 | 1.98E-10 | 2.20228E-09 | NADH dehydrogenase |
| WP_060382429.1 | -1.91635 | 8.841991 | 8.35E-12 | 1.12941E-10 | MBL fold metallo-hydrolase |
| WP_060381304.1 | -1.908793 | 2.272921 | 8.25E-10 | 8.11119E-09 | transposase |
| WP_060383512.1 | -1.900919 | 2.557358 | 1.60E-08 | 1.23572E-07 | hypothetical protein |
| WP_060382408.1 | -1.893039 | 8.942041 | 5.66E-06 | 2.47165E-05 | coproporphyrinogen III oxidase |
| WP_060383213.1 | -1.889325 | 3.68364 | 0.000492 | 0.001362184 | hypothetical protein |
| WP_060381432.1 | -1.873809 | 5.637942 | 6.91E-06 | 2.95684E-05 | hypothetical protein |
| WP_060381906.1 | -1.859077 | 3.317949 | 2.02E-05 | 7.82055E-05 | hypothetical protein |
| WP_060381565.1 | -1.853575 | 6.635055 | 6.51E-10 | 6.60524E-09 | NADH-quinone oxidoreductase subunit K |
| WP_060381422.1 | -1.8492 | 8.059588 | 5.41E-11 | 6.4874E-10 | four helix bundle protein |
| WP_060383259.1 | -1.847806 | 13.11652 | 1.10E-07 | 6.87264E-07 | gliding motility protein GldN |
| WP_060381564.1 | -1.839845 | 7.447729 | 2.88E-10 | 3.13669E-09 | NADH dehydrogenase |
| WP_060383834.1 | -1.834672 | 5.675174 | 2.28E-18 | 6.6715E-17 | alpha/beta hydrolase |
| WP_060381304.1 | -1.828619 | 2.140486 | 2.38E-11 | 2.98501E-10 | transposase |
| WP_060382024.1 | -1.818861 | 8.13202 | 7.63E-12 | 1.05538E-10 | DEAD/DEAH box helicase |
| WP_060383120.1 | -1.788959 | 8.671223 | 5.84E-08 | 3.91063E-07 | recombination protein RecR |
| WP_060383512.1 | -1.788438 | 2.491168 | 1.77E-05 | 6.94728E-05 | hypothetical protein |
| WP_060383216.1 | -1.770376 | 4.599132 | 2.21E-07 | 1.28175E-06 | epimerase |
| WP_060382936.1 | -1.744034 | 6.960098 | 1.87E-05 | 7.26178E-05 | nitrous oxide reductase |
| WP_060383513.1 | -1.741659 | 2.690376 | 2.18E-08 | 1.60487E-07 | hypothetical protein |
| WP_060381556.1 | -1.714178 | 7.947913 | 2.14E-09 | 1.94077E-08 | NADH dehydrogenase |
| WP_060382922.1 | -1.711919 | 9.165178 | 0.001219 | 0.003046905 | hypothetical protein |
| WP_060383119.1 | -1.693837 | 8.639823 | 1.43E-06 | 7.09189E-06 | sugar transporter |
| WP_060383513.1 | -1.681946 | 2.727149 | 5.01E-08 | 3.42722E-07 | hypothetical protein |
| WP_060381905.1 | -1.671569 | 5.065671 | 6.40E-06 | 2.75216E-05 | hypothetical protein |
| WP_060383400.1 | -1.670649 | 2.093476 | 3.21E-07 | 1.80065E-06 | transposase |
| WP_060383835.1 | -1.666089 | 7.324971 | 4.02E-06 | 1.82119E-05 | type I glyceraldehyde-3-phosphate dehydrogenase |
| WP_060382474.1 | -1.663368 | 6.136303 | 0.003417 | 0.007697286 | hypothetical protein |
| WP_060383402.1 | -1.658737 | 9.053654 | 2.45E-12 | 3.66725E-11 | 9SS C-terminal target domain-containing protein |
| WP_060381304.1 | -1.653374 | 2.241442 | 3.40E-07 | 1.8934E-06 | transposase |
| WP_060382920.1 | -1.650533 | 9.592328 | 0.004213 | 0.009290294 | hypothetical protein |
| WP_060382430.1 | -1.625703 | 7.949392 | 3.31E-07 | 1.84483E-06 | MBL fold metallo-hydrolase |
| WP_060381594.1 | -1.625615 | 7.30669 | 2.84E-08 | 2.04929E-07 | hypothetical protein |
| WP_060381368.1 | -1.624108 | 6.466952 | 1.13E-07 | 7.02347E-07 | glycosyltransferase |
| WP_060381568.1 | -1.605561 | 8.609743 | 1.87E-08 | 1.40487E-07 | ADH-quinone oxidoreductase subunit N |
| WP_060381621.1 | -1.595663 | 6.020889 | 1.74E-08 | 1.3291E-07 | hypothetical protein |
| WP_060382078.1 | -1.561943 | 8.281578 | 2.71E-08 | 1.96063E-07 | 6-phosphofructokinase |
| WP_060381567.1 | -1.520794 | 8.474168 | 1.12E-10 | 1.27044E-09 | NADH-quinone oxidoreductase subunit M |
| WP_060381582.1 | -1.511064 | 8.334818 | 3.45E-06 | 1.57688E-05 | cadmium/zinc/cobalt-transporting ATPase |
| WP_060382776.1 | -1.510309 | 9.777515 | 0.009501 | 0.019002887 | cadmium/zinc/cobalt-transporting ATPase |
| WP_060383671.1 | -1.503533 | 8.462906 | 1.11E-08 | 8.89165E-08 | succinate dehydrogenase |
| WP_060382427.1 | -1.489861 | 6.178942 | 3.16E-08 | 2.26205E-07 | thioredoxin |
| WP_060382250.1 | -1.488945 | 7.310715 | 1.62E-05 | 6.42441E-05 | hybrid sensor histidine kinase/response regulator |
| WP_060382937.1 | -1.478816 | 5.305044 | 0.000149 | 0.000467988 | cytochrome C |
| WP_060382161.1 | -1.46725 | 5.562705 | 2.39E-13 | 4.04374E-12 | nitric oxide reductase |
| WP_060381703.1 | -1.466216 | 4.619485 | 6.63E-14 | 1.26343E-12 | histidine kinase |
| WP_060382919.1 | -1.412753 | 7.588117 | 0.000606 | 0.001630766 | cytochrome-c peroxidase |
| WP_060381435.1 | -1.407257 | 8.544385 | 0.000604 | 0.001629584 | phosphoribosylpyrophosphate synthetase |
| WP_060382144.1 | -1.402535 | 7.612485 | 0.000259 | 0.000762225 | hypothetical protein |
| WP_060381947.1 | -1.393992 | 4.027706 | 1.71E-13 | 3.00477E-12 | 2-succinyl-6-hydroxy-2,4-cyclohexadiene-1-carboxylate synthase |
| WP_060383513.1 | -1.38489 | 2.703515 | 3.91E-05 | 0.000142911 | hypothetical protein |
| WP_060382308.1 | -1.37565 | 0.094007 | 0.002218 | 0.005190161 | hypothetical protein |
| WP_060382620.1 | -1.348925 | 7.497128 | 1.37E-13 | 2.44862E-12 | cold-shock protein |
| WP_060382910.1 | -1.341642 | 12.58366 | 6.02E-07 | 3.24662E-06 | hypothetical protein |
| WP_060383542.1 | -1.327635 | 4.328718 | 9.40E-10 | 9.09446E-09 | hypothetical protein |
| WP_060382361.1 | -1.318608 | 5.752111 | 8.46E-08 | 5.41774E-07 | hypothetical protein |
| WP_060383744.1 | -1.316429 | 8.314074 | 7.19E-08 | 4.71158E-07 | cobalt transporter |
| WP_060381284.1 | -1.315386 | 6.226956 | 1.23E-07 | 7.52537E-07 | hypothetical protein |
| WP_060381798.1 | -1.315186 | 5.827315 | 6.12E-07 | 3.2814E-06 | hypothetical protein |
| WP_060383801.1 | -1.313577 | 6.623528 | 6.46E-08 | 4.28836E-07 | L-aspartate oxidase |
| WP_060383282.1 | -1.303966 | 9.976461 | 1.23E-05 | 4.95673E-05 | penicillin-binding protein |
| WP_060382249.1 | -1.297164 | 8.042629 | 1.47E-11 | 1.90721E-10 | purine-nucleoside phosphorylase |
| WP_060383195.1 | -1.278343 | 10.81256 | 7.29E-05 | 0.000248563 | hypothetical protein |
| WP_060383118.1 | -1.264139 | 8.518281 | 1.40E-05 | 5.61277E-05 | sugar transporter |
| WP_060381797.1 | -1.252984 | 6.051312 | 0.000487 | 0.001348684 | sulfate transporter |
| WP_060381552.1 | -1.234786 | 3.404309 | 4.09E-09 | 3.51631E-08 | A/G-specific adenine glycosylase |
| WP_060383758.1 | -1.232117 | 2.858852 | 1.19E-06 | 6.02645E-06 | N-acetyl-gamma-glutamyl-phosphate reductase |
| WP_060383278.1 | -1.221734 | 9.568864 | 3.04E-07 | 1.71139E-06 | cell division protein FtsW |
| WP_060382307.1 | -1.204841 | 5.69098 | 7.09E-08 | 4.67167E-07 | hypothetical protein |
| WP_060382946.1 | -1.204169 | 5.893691 | 4.82E-05 | 0.000172034 | globin |
| WP_060383686.1 | -1.199552 | 8.76514 | 1.76E-05 | 6.92168E-05 | tRNA dihydrouridine synthase DusB |
| WP_060382656.1 | -1.19882 | 8.538117 | 1.09E-07 | 6.80834E-07 | hypothetical protein |
| WP_060382310.1 | -1.19591 | 6.222047 | 3.28E-08 | 2.32694E-07 | hypothetical protein |
| WP_060383789.1 | -1.187911 | -0.18406 | 0.017521 | 0.032295897 | hypothetical protein |
| WP_060383323.1 | -1.18737 | 6.374472 | 5.99E-15 | 1.32206E-13 | hypothetical protein |
| WP_060382998.1 | -1.184098 | 6.140559 | 2.57E-09 | 2.29046E-08 | alanine--tRNA ligase |
| WP_060382935.1 | -1.180657 | 3.521961 | 0.000352 | 0.001016504 | hypothetical protein |
| WP_060381725.1 | -1.176938 | 4.046021 | 1.97E-06 | 9.53692E-06 | amino acid transporter |
| WP_060381423.1 | -1.172988 | 7.485395 | 5.64E-07 | 3.05234E-06 | nicotinic acid mononucleotide adenylyltransferase |
| WP_060382428.1 | -1.169832 | 5.138547 | 2.95E-07 | 1.67781E-06 | hypothetical protein |
| WP_060381807.1 | -1.165499 | 7.489789 | 4.79E-09 | 4.04745E-08 | beta-ketoacyl-ACP reductase |
| WP_060381581.1 | -1.160533 | 7.727658 | 0.000389 | 0.001111381 | efflux transporter periplasmic adaptor subunit |
| WP_060382306.1 | -1.160508 | 7.739562 | 3.71E-08 | 2.59963E-07 | hypothetical protein |
| WP_060381421.1 | -1.153679 | 7.662772 | 1.33E-13 | 2.40616E-12 | guanylate kinase |
| WP_060382513.1 | -1.148376 | 8.634755 | 4.40E-05 | 0.000158552 | replicative DNA helicase |
| WP_060381876.1 | -1.148191 | 7.609694 | 2.93E-10 | 3.17455E-09 | hypothetical protein |
| WP_060382589.1 | -1.146431 | 9.17831 | 1.33E-06 | 6.63848E-06 | methylcrotonoyl-CoA carboxylase |
| WP_060381401.1 | -1.143958 | 11.83408 | 1.17E-05 | 4.7354E-05 | hypothetical protein |
| WP_060382657.1 | -1.127396 | 7.934197 | 7.93E-05 | 0.000267643 | MATE family efflux transporter |
| WP_060381350.1 | -1.122055 | 8.495761 | 4.78E-10 | 4.94607E-09 | ATP synthase subunit delta |
| WP_060382784.1 | -1.121906 | 6.855815 | 5.23E-13 | 8.60129E-12 | mannose-6-phosphate isomerase |
| WP_060382309.1 | -1.109351 | 4.63153 | 1.17E-06 | 5.94356E-06 | hypothetical protein |
| WP_060383138.1 | -1.108646 | 5.431245 | 2.85E-05 | 0.000106755 | hypothetical protein |
| WP_060382119.1 | -1.106985 | 10.65154 | 0.000399 | 0.001132309 | histidine kinase |
| WP_060383794.1 | -1.099263 | 9.526734 | 1.49E-05 | 5.94656E-05 | heat-shock protein Hsp70 |
| WP_060381812.1 | -1.099024 | 5.466778 | 1.74E-08 | 1.3291E-07 | hypothetical protein |
| WP_060383698.1 | -1.068242 | 5.776374 | 4.64E-09 | 3.94176E-08 | hypothetical protein |
| WP_060383465.1 | -1.059399 | 5.012751 | 1.38E-12 | 2.1765E-11 | epoxyqueuosine reductase |
| WP_060381710.1 | -1.05882 | 7.222792 | 7.61E-06 | 3.2332E-05 | hypothetical protein |
| WP_060382079.1 | -1.057978 | 9.095406 | 2.35E-05 | 8.93149E-05 | type I glyceraldehyde-3-phosphate dehydrogenase |
| WP_060383491.1 | -1.056217 | 7.55332 | 2.99E-10 | 3.21048E-09 | 16S rRNA processing protein RimM |
| WP_060383281.1 | -1.056136 | 9.441785 | 4.40E-06 | 1.98209E-05 | UDP-N-acetylmuramoyl-L-alanyl-D-glutamate--2,6-diaminopimelate ligase |
| WP_060381332.1 | -1.055137 | 7.670179 | 6.99E-06 | 2.98623E-05 | NAD(P)-dependent oxidoreductase |
| WP_060382567.1 | -1.053801 | 9.141671 | 4.03E-10 | 4.24542E-09 | endopeptidase La |
| WP_060383532.1 | -1.043204 | 10.06982 | 1.17E-05 | 4.74841E-05 | acyl-CoA dehydrogenase |
| WP_060383134.1 | -1.04099 | 2.895616 | 0.000422 | 0.001189076 | hypothetical protein |
| WP_060382933.1 | -1.036939 | 4.24678 | 5.27E-05 | 0.000185688 | nitrous oxide reductase |
| WP_060382403.1 | -1.034044 | 7.41083 | 6.84E-05 | 0.000235634 | translation factor Sua5 |
| WP_014165133.1 | -1.03391 | 7.109017 | 2.21E-14 | 4.58732E-13 | acyl-CoA thioesterase |
| WP_060383442.1 | -1.029356 | 7.906772 | 1.83E-12 | 2.83135E-11 | protoheme IX farnesyltransferase |
| WP_060381736.1 | -1.028362 | 8.632304 | 0.000533 | 0.001461417 | methylmalonyl-CoA mutase |
| WP_060383852.1 | -1.01456 | 9.273206 | 2.61E-09 | 2.31917E-08 | branched-chain amino acid aminotransferase |
| WP_060383115.1 | -1.013068 | 8.776242 | 2.15E-10 | 2.37189E-09 | UDP-glucose 6-dehydrogenase |
| WP_060381445.1 | -1.009919 | 7.787501 | 3.74E-10 | 3.95515E-09 | glycerol-3-phosphate dehydrogenase |
| WP_060382483.1 | -1.007693 | 8.04533 | 0.00078 | 0.002049917 | cytochrome C oxidase Cbb3 |
| WP_060381348.1 | -1.000604 | 9.431175 | 7.40E-08 | 4.82557E-07 | ATP synthase F0 subunit A |
| WP_060381597.1 | -1.000211 | 6.140282 | 3.68E-08 | 2.5867E-07 | SAM-dependent methyltransferase |
| WP_060382398.1 | 1.0001 | 3.903902 | 1.11E-07 | 6.94001E-07 | nucleoside-diphosphate sugar epimerase |
| WP_060383636.1 | 1.00314 | 5.277927 | 6.40E-05 | 0.000221874 | hypothetical protein |
| WP_060382525.1 | 1.00802 | 7.879043 | 0.000613 | 0.001645064 | DNA-binding protein |
| WP_060382696.1 | 1.01296 | 4.770602 | 7.96E-08 | 5.13438E-07 | thiol:disulfide interchange protein |
| WP_060383848.1 | 1.01663 | 3.162641 | 0.000181 | 0.000554161 | hypothetical protein |
| WP_060381323.1 | 1.01857 | 6.568607 | 3.67E-09 | 3.1726E-08 | hypothetical protein |
| WP_060381484.1 | 1.01866 | 7.540613 | 9.90E-09 | 8.03369E-08 | AAA family ATPase |
| WP_060383880.1 | 1.02148 | 5.345678 | 2.11E-09 | 1.91455E-08 | DUF4442 domain-containing protein |
| WP_060382764.1 | 1.02398 | 10.7711 | 2.31E-11 | 2.91193E-10 | collagen-binding protein |
| WP_060383321.1 | 1.02515 | 3.983121 | 4.60E-06 | 2.05912E-05 | hypothetical protein |
| WP_060381375.1 | 1.02705 | 6.180321 | 9.20E-07 | 4.79421E-06 | hypothetical protein |
| WP_060382014.1 | 1.02777 | 6.07398 | 1.07E-12 | 1.711E-11 | NAD-dependent deacylase |
| WP_060381867.1 | 1.02857 | 7.79404 | 1.91E-09 | 1.75105E-08 | hypothetical protein |
| WP_060381992.1 | 1.03204 | 2.972331 | 4.24E-05 | 0.00015302 | hypothetical protein |
| WP_060383793.1 | 1.0367 | 5.228405 | 4.05E-07 | 2.23292E-06 | ABC transporter permease |
| WP_060383422.1 | 1.03969 | 7.259304 | 5.97E-09 | 4.98754E-08 | hypothetical protein |
| WP_060382583.1 | 1.04278 | 5.564037 | 4.78E-10 | 4.94607E-09 | excinuclease ABC subunit B |
| WP_060383297.1 | 1.04466 | 10.44064 | 2.00E-07 | 1.17961E-06 | hypothetical protein |
| WP_060381976.1 | 1.04648 | 6.765991 | 3.93E-12 | 5.68316E-11 | hypothetical protein |
| WP_060383423.1 | 1.0504 | 7.645461 | 1.08E-10 | 1.23162E-09 | di-trans-poly-cis-decaprenylcistransferase |
| WP_060382391.1 | 1.05244 | 7.525576 | 1.12E-06 | 5.70885E-06 | hypothetical protein |
| WP_060382580.1 | 1.05383 | 7.210313 | 4.75E-18 | 1.31444E-16 | non-canonical purine NTP diphosphatase |
| WP_060383537.1 | 1.05403 | 5.026086 | 2.89E-06 | 1.34494E-05 | hypothetical protein |
| WP_060383425.1 | 1.05824 | 8.386037 | 5.49E-12 | 7.70354E-11 | hypothetical protein |
| WP_060383824.1 | 1.05868 | 4.920881 | 6.49E-09 | 5.40914E-08 | hypothetical protein |
| WP_060381973.1 | 1.05934 | 4.297534 | 4.32E-08 | 2.98658E-07 | catalase |
| WP_060383025.1 | 1.06047 | 4.208032 | 6.23E-06 | 2.69274E-05 | transcription factor |
| WP_060381321.1 | 1.06102 | 5.877064 | 7.83E-12 | 1.07621E-10 | hypothetical protein |
| WP_060382503.1 | 1.0669 | 2.607642 | 4.10E-05 | 0.000148887 | hypothetical protein |
| WP_060383467.1 | 1.07151 | 6.129641 | 1.72E-08 | 1.32109E-07 | amino acid transporter |
| WP_060382631.1 | 1.07353 | 4.547518 | 1.14E-07 | 7.02439E-07 | hypothetical protein |
| WP_060381391.1 | 1.07389 | 3.370628 | 8.68E-06 | 3.66595E-05 | hypothetical protein |
| WP_060381282.1 | 1.07748 | 3.782906 | 0.000443 | 0.001235846 | hypothetical protein |
| WP_060382768.1 | 1.07836 | 3.623763 | 5.23E-05 | 0.000184451 | hypothetical protein |
| WP_060383476.1 | 1.08084 | 6.547644 | 1.80E-08 | 1.36103E-07 | hypothetical protein |
| WP_060382372.1 | 1.0898 | 6.919013 | 1.97E-09 | 1.79236E-08 | 3-methyl-2-oxobutanoate hydroxymethyltransferase |
| WP_060382384.1 | 1.09421 | 10.02809 | 1.88E-09 | 1.72935E-08 | two-component system response regulator |
| WP_060383123.1 | 1.09484 | 7.979734 | 7.08E-11 | 8.3677E-10 | CoA-binding protein |
| WP_060382901.1 | 1.09494 | 10.66146 | 1.20E-08 | 9.48912E-08 | RNA polymerase subunit sigma-54 |
| WP_060382855.1 | 1.09583 | 9.033507 | 1.77E-11 | 2.26683E-10 | ABC transporter permease |
| WP_060382791.1 | 1.09838 | 7.564899 | 3.18E-08 | 2.26972E-07 | hypothetical protein |
| WP_060381611.1 | 1.09845 | 3.978543 | 4.08E-08 | 2.84255E-07 | hypothetical protein |
| WP_060382697.1 | 1.10236 | 8.639614 | 1.09E-07 | 6.80834E-07 | hypothetical protein |
| WP_060382328.1 | 1.1056 | 3.298001 | 9.40E-08 | 5.9562E-07 | hypothetical protein |
| WP_060381612.1 | 1.10755 | 3.534459 | 4.42E-07 | 2.42484E-06 | hypothetical protein |
| WP_060381392.1 | 1.11239 | 3.139791 | 1.78E-05 | 6.94728E-05 | hypothetical protein |
| WP_060383055.1 | 1.115 | 8.973258 | 8.57E-10 | 8.39367E-09 | hypothetical protein |
| WP_060381443.1 | 1.11995 | 12.23509 | 6.03E-07 | 3.24662E-06 | NADH-dependent alcohol dehydrogenase |
| WP_060382658.1 | 1.12019 | 9.216781 | 3.62E-07 | 2.00512E-06 | hypothetical protein |
| WP_060382485.1 | 1.12293 | 6.73871 | 1.77E-18 | 5.23324E-17 | Crp/Fnr family transcriptional regulator |
| WP_060383708.1 | 1.12315 | 3.309199 | 8.57E-08 | 5.45829E-07 | thiamine biosynthesis protein ThiS |
| WP_060381406.1 | 1.12452 | 6.324629 | 2.86E-09 | 2.48695E-08 | helicase |
| WP_060383729.1 | 1.12455 | 7.360235 | 1.23E-08 | 9.67439E-08 | hypothetical protein |
| WP_060382831.1 | 1.12815 | 9.270386 | 5.07E-10 | 5.22543E-09 | phosphoglucomutase |
| WP_060382837.1 | 1.13269 | 6.175612 | 1.84E-13 | 3.22361E-12 | cell division protein ZapA |
| WP_060382397.1 | 1.1331 | 4.77886 | 1.00E-11 | 1.3337E-10 | hypothetical protein |
| WP_060382062.1 | 1.13479 | 7.228906 | 5.24E-17 | 1.31851E-15 | osmotically inducible protein OsmC |
| WP_060381476.1 | 1.13649 | 7.151103 | 1.38E-12 | 2.1765E-11 | hypothetical protein |
| WP_060382497.1 | 1.14011 | 7.487133 | 4.63E-15 | 1.03128E-13 | hypothetical protein |
| WP_060381283.1 | 1.1409 | 6.292429 | 7.28E-10 | 7.29521E-09 | single-stranded DNA-binding protein |
| WP_060382499.1 | 1.14458 | 5.457566 | 4.90E-08 | 3.35746E-07 | permease |
| WP_060382949.1 | 1.14666 | 6.68035 | 3.62E-12 | 5.29478E-11 | hypothetical protein |
| WP_060382700.1 | 1.15307 | 5.543428 | 0.000534 | 0.001464284 | cob(I)yrinic acid a c-diamide adenosyltransferase |
| WP_060382578.1 | 1.15425 | 7.302337 | 7.96E-11 | 9.27296E-10 | hypothetical protein |
| WP_060381609.1 | 1.15584 | 3.767067 | 2.33E-09 | 2.08943E-08 | hypothetical protein |
| WP_060383667.1 | 1.15872 | 7.684975 | 7.62E-09 | 6.30924E-08 | hypothetical protein |
| WP_060381383.1 | 1.1707 | 10.28724 | 5.81E-09 | 4.87436E-08 | hypothetical protein |
| WP_060383737.1 | 1.17142 | 5.738647 | 3.01E-07 | 1.70431E-06 | dihydroneopterin aldolase |
| WP_060383199.1 | 1.17385 | 4.355146 | 6.96E-10 | 7.00631E-09 | hypothetical protein |
| WP_060383812.1 | 1.17532 | 6.593668 | 3.66E-16 | 8.6068E-15 | transposase |
| WP_060382216.1 | 1.18285 | 4.895438 | 1.21E-08 | 9.49636E-08 | hypothetical protein |
| WP_060382047.1 | 1.19804 | 5.59251 | 3.10E-11 | 3.85696E-10 | Crp/Fnr family transcriptional regulator |
| WP_060382943.1 | 1.19948 | 4.225066 | 4.79E-09 | 4.04745E-08 | hypothetical protein |
| WP_060383124.1 | 1.20764 | 8.360609 | 5.10E-12 | 7.24556E-11 | antibiotic resistance protein MarC |
| WP_060382763.1 | 1.21126 | 9.347635 | 1.21E-15 | 2.80777E-14 | hypothetical protein |
| WP_060383581.1 | 1.21404 | 6.941826 | 4.41E-11 | 5.38233E-10 | hydroxyacid dehydrogenase |
| WP_060381662.1 | 1.21644 | 6.351329 | 3.90E-12 | 5.66722E-11 | hypothetical protein |
| WP_060381931.1 | 1.2212 | 4.356933 | 4.01E-07 | 2.21775E-06 | transcriptional regulator |
| WP_060383825.1 | 1.22148 | 7.731181 | 0.000217 | 0.000651194 | muramidase |
| WP_014164231.1 | 1.22157 | 8.271343 | 9.60E-07 | 4.97757E-06 | hypothetical protein |
| WP_060382522.1 | 1.22586 | 9.083321 | 2.18E-13 | 3.72502E-12 | hypothetical protein |
| WP_060383507.1 | 1.22672 | 7.304264 | 1.74E-22 | 7.16266E-21 | growth inhibitor PemK |
| WP_060381769.1 | 1.22976 | 5.985744 | 5.37E-11 | 6.46854E-10 | hypothetical protein |
| WP_060382705.1 | 1.23068 | 3.930094 | 0.000123 | 0.000394151 | hypothetical protein |
| WP_060382957.1 | 1.2332 | 4.485988 | 2.01E-06 | 9.66439E-06 | hypothetical protein |
| WP_060383904.1 | 1.23949 | 6.022379 | 3.33E-08 | 2.3566E-07 | 3\'-5\' exonuclease |
| WP_060381635.1 | 1.24053 | 1.851533 | 0.000174 | 0.000536626 | hypothetical protein |
| WP_060381933.1 | 1.24735 | 8.828876 | 1.68E-13 | 2.98462E-12 | hypothetical protein |
| WP_060383738.1 | 1.25047 | 6.290713 | 2.04E-08 | 1.51466E-07 | hypothetical protein |
| WP_060382371.1 | 1.2518 | 6.818551 | 1.09E-09 | 1.04242E-08 | RNA pseudouridine synthase |
| WP_060381777.1 | 1.25461 | 1.141965 | 4.22E-05 | 0.000152654 | hypothetical protein |
| WP_060381478.1 | 1.25485 | 9.132584 | 5.50E-14 | 1.08939E-12 | molecular chaperone DnaK |
| WP_060381504.1 | 1.25637 | 8.476336 | 8.65E-07 | 4.54417E-06 | hypothetical protein |
| WP_060381844.1 | 1.25863 | 5.216404 | 3.58E-13 | 5.93184E-12 | phosphinothricin acetyltransferase |
| WP_060383181.1 | 1.26021 | 4.653099 | 6.50E-14 | 1.24836E-12 | hypothetical protein |
| WP_060383292.1 | 1.26051 | 7.077082 | 8.38E-19 | 2.54037E-17 | helix-turn-helix transcriptional regulator |
| WP_060383719.1 | 1.26711 | 6.929839 | 1.89E-17 | 5.00745E-16 | GNAT family acetyltransferase |
| WP_060383847.1 | 1.26866 | 4.135706 | 3.23E-10 | 3.45555E-09 | hypothetical protein |
| WP_060382366.1 | 1.27124 | 6.787778 | 4.22E-09 | 3.608E-08 | hypothetical protein |
| WP_060383906.1 | 1.27509 | 4.388141 | 3.25E-06 | 1.49589E-05 | hypothetical protein |
| WP_060383550.1 | 1.27537 | 3.830284 | 9.34E-05 | 0.000310336 | 1-acyl-sn-glycerol-3-phosphate acyltransferase |
| WP_060383504.1 | 1.27704 | 4.785884 | 5.30E-08 | 3.58669E-07 | methylmalonyl-CoA epimerase |
| WP_060382041.1 | 1.27827 | 8.223193 | 4.86E-11 | 5.87741E-10 | hypothetical protein |
| WP_060383180.1 | 1.28335 | 6.141368 | 5.88E-14 | 1.14753E-12 | ABC transporter ATP-binding protein |
| WP_060381727.1 | 1.28651 | 6.523406 | 1.34E-07 | 8.16587E-07 | RNA-splicing ligase RtcB |
| WP_060381633.1 | 1.29538 | 8.897917 | 2.10E-14 | 4.40642E-13 | hypothetical protein |
| WP_060381449.1 | 1.30066 | 5.378455 | 1.04E-06 | 5.34294E-06 | colicin V production protein |
| WP_060381975.1 | 1.30256 | 9.415081 | 1.34E-13 | 2.40616E-12 | alkyl hydroperoxide reductase |
| WP_060382539.1 | 1.30653 | 6.698195 | 4.44E-11 | 5.39916E-10 | DUF4834 domain-containing protein |
| WP_060381757.1 | 1.31041 | 8.590069 | 1.68E-10 | 1.89341E-09 | hypothetical protein |
| WP_060382470.1 | 1.3108 | 5.952907 | 1.05E-10 | 1.20522E-09 | 23S rRNA (pseudouridine(1915)-N(3))-methyltransferase RlmH |
| WP_060382368.1 | 1.31141 | 12.19271 | 2.35E-09 | 2.10095E-08 | glutamine synthetase |
| WP_060382830.1 | 1.32016 | 8.770585 | 2.39E-12 | 3.60614E-11 | glycosyl transferase family 2 |
| WP_060382739.1 | 1.32538 | 8.702217 | 3.18E-08 | 2.26972E-07 | thioredoxin |
| WP_060383089.1 | 1.34029 | 5.285435 | 3.34E-09 | 2.8989E-08 | ABC transporter permease |
| WP_060382009.1 | 1.34177 | 7.183717 | 1.19E-17 | 3.17448E-16 | hypothetical protein |
| WP_060381978.1 | 1.34766 | 4.374454 | 2.16E-13 | 3.72502E-12 | SAM-dependent methyltransferase |
| WP_060381374.1 | 1.35125 | 8.876835 | 5.55E-14 | 1.09034E-12 | ferredoxin--NADP(+) reductase |
| WP_060381913.1 | 1.36095 | 8.304588 | 3.23E-13 | 5.38367E-12 | hypothetical protein |
| WP_060383426.1 | 1.36603 | 9.94089 | 5.98E-11 | 7.13363E-10 | hypothetical protein |
| WP_060381770.1 | 1.36905 | 6.315897 | 5.05E-12 | 7.21856E-11 | RNA-binding protein |
| WP_060381785.1 | 1.36911 | 3.042683 | 5.55E-07 | 3.01352E-06 | hypothetical protein |
| WP_060382840.1 | 1.37398 | 8.299183 | 1.20E-07 | 7.36797E-07 | hypothetical protein |
| WP_060381968.1 | 1.37806 | 5.890936 | 8.54E-13 | 1.37692E-11 | transcriptional regulator |
| WP_060381606.1 | 1.3785 | 4.582837 | 1.31E-08 | 1.01759E-07 | hypothetical protein |
| WP_060382678.1 | 1.39485 | 7.949126 | 1.81E-10 | 2.03566E-09 | hypothetical protein |
| WP_060382126.1 | 1.39802 | 2.056752 | 1.79E-06 | 8.74338E-06 | restriction endonuclease subunit R |
| WP_060383420.1 | 1.40488 | 6.173865 | 7.67E-10 | 7.63249E-09 | hypothetical protein |
| WP_060382599.1 | 1.40627 | 7.855393 | 9.22E-14 | 1.6926E-12 | nucleoside triphosphate pyrophosphohydrolase |
| WP_060381746.1 | 1.41584 | 7.380702 | 6.88E-14 | 1.30068E-12 | hypothetical protein |
| WP_060383222.1 | 1.41757 | 6.678646 | 3.66E-11 | 4.49131E-10 | 3-oxoacyl-ACP synthase |
| WP_060382868.1 | 1.42081 | 11.02866 | 1.03E-11 | 1.36278E-10 | serine protease |
| WP_060382871.1 | 1.43104 | 9.506231 | 1.05E-10 | 1.20522E-09 | acetyltransferase |
| WP_060381383.1 | 1.43227 | 8.912427 | 2.11E-13 | 3.66755E-12 | hypothetical protein |
| WP_060383387.1 | 1.44258 | 4.876466 | 3.60E-11 | 4.437E-10 | hypothetical protein |
| WP_060382137.1 | 1.44556 | 3.489552 | 2.36E-05 | 8.94358E-05 | RNA polymerase subunit sigma-70 |
| WP_060381551.1 | 1.45608 | 7.118773 | 4.77E-20 | 1.57834E-18 | single-stranded DNA-binding protein |
| WP_060383369.1 | 1.48222 | 8.702484 | 3.61E-07 | 2.00279E-06 | thioredoxin |
| WP_060382630.1 | 1.48306 | 6.223989 | 1.26E-21 | 4.8222E-20 | hypothetical protein |
| WP_060383601.1 | 1.48611 | 3.892673 | 2.33E-06 | 1.10895E-05 | hypothetical protein |
| WP_060383248.1 | 1.48628 | 4.680687 | 2.01E-10 | 2.22571E-09 | AsnC family transcriptional regulator |
| WP_060382508.1 | 1.49391 | 1.486251 | 1.06E-06 | 5.41403E-06 | hypothetical protein |
| WP_060381929.1 | 1.49684 | 3.244918 | 1.48E-06 | 7.3403E-06 | hypothetical protein |
| WP_060383036.1 | 1.50869 | 10.21155 | 1.02E-06 | 5.27119E-06 | cyclase |
| WP_060382414.1 | 1.52169 | 4.94919 | 1.11E-12 | 1.76564E-11 | AsnC family transcriptional regulator |
| WP_060383765.1 | 1.52298 | 6.64372 | 8.09E-12 | 1.10661E-10 | hypothetical protein |
| WP_060383689.1 | 1.52566 | 6.136414 | 1.43E-16 | 3.4211E-15 | transcriptional regulator |
| WP_060382110.1 | 1.5301 | 4.646673 | 2.65E-09 | 2.33084E-08 | transcriptional regulator |
| WP_060383656.1 | 1.53161 | 7.805838 | 9.80E-11 | 1.13592E-09 | ferrous iron transport protein B |
| WP_060382373.1 | 1.53433 | 6.785074 | 1.96E-20 | 6.75822E-19 | large-conductance mechanosensitive channel |
| WP_060381925.1 | 1.53483 | 4.415061 | 8.12E-09 | 6.68051E-08 | hypothetical protein |
| WP_060383791.1 | 1.54399 | 5.875844 | 2.34E-18 | 6.73394E-17 | hypothetical protein |
| WP_060382067.1 | 1.55715 | 4.920698 | 7.02E-17 | 1.71401E-15 | hypothetical protein |
| WP_060381583.1 | 1.55926 | 7.692781 | 3.82E-17 | 9.70871E-16 | hypothetical protein |
| WP_060381932.1 | 1.56682 | 8.707618 | 6.71E-15 | 1.46894E-13 | hypothetical protein |
| WP_060383854.1 | 1.57043 | 6.147511 | 1.66E-11 | 2.13727E-10 | aquaporin |
| WP_060383326.1 | 1.57479 | 10.73342 | 1.16E-08 | 9.18724E-08 | electron transfer flavoprotein subunit alpha |
| WP_060383877.1 | 1.57747 | 4.78133 | 9.49E-17 | 2.29626E-15 | methanol dehydrogenase |
| WP_060382836.1 | 1.58433 | 6.174188 | 3.73E-23 | 1.64607E-21 | hypothetical protein |
| WP_060383810.1 | 1.58492 | 1.835 | 8.23E-07 | 4.36778E-06 | hypothetical protein |
| WP_060382577.1 | 1.58826 | 10.58507 | 1.92E-07 | 1.1318E-06 | TonB-dependent receptor |
| WP_060381919.1 | 1.59176 | 7.678662 | 3.46E-22 | 1.38204E-20 | O-succinylbenzoic acid--CoA ligase |
| WP_060382997.1 | 1.59819 | 6.389905 | 4.10E-14 | 8.24393E-13 | peptidase M23 |
| WP_060382969.1 | 1.61315 | 1.624822 | 5.47E-08 | 3.68167E-07 | cupin |
| WP_060382396.1 | 1.61964 | 5.57892 | 4.91E-14 | 9.80812E-13 | hypothetical protein |
| WP_060381311.1 | 1.62981 | 8.660461 | 7.86E-14 | 1.47653E-12 | histidine kinase |
| WP_060381599.1 | 1.63507 | 6.844685 | 1.50E-15 | 3.40189E-14 | DUF5103 domain-containing protein |
| WP_060381309.1 | 1.64115 | 9.115084 | 6.84E-13 | 1.10969E-11 | T9SS C-terminal target domain-containing protein |
| WP_060381969.1 | 1.64849 | 6.949804 | 1.08E-31 | 9.06994E-30 | NAD(P)H-dependent oxidoreductase |
| WP_060382993.1 | 1.64938 | 5.870052 | 9.49E-22 | 3.67415E-20 | hypothetical protein |
| WP_014165796.1 | 1.66547 | 4.357067 | 3.16E-11 | 3.91732E-10 | hypothetical protein |
| WP_060382587.1 | 1.66579 | 10.5364 | 2.31E-09 | 2.08424E-08 | phosphate transporter |
| WP_060383589.1 | 1.67235 | 9.676746 | 2.18E-17 | 5.7042E-16 | glutamyl-tRNA reductase |
| WP_060381979.1 | 1.68328 | 3.807785 | 2.56E-14 | 5.23883E-13 | hypothetical protein |
| WP_060381837.1 | 1.6861 | 3.780971 | 3.17E-12 | 4.69796E-11 | MarR family transcriptional regulator |
| WP_060383040.1 | 1.68693 | 7.133548 | 0.00017 | 0.000525642 | nitric oxide dioxygenase |
| WP_060381679.1 | 1.69292 | 2.476874 | 1.20E-06 | 6.03279E-06 | hypothetical protein |
| WP_060383910.1 | 1.69973 | 5.996754 | 2.26E-16 | 5.35593E-15 | hypothetical protein |
| WP_060382390.1 | 1.70208 | 7.862525 | 1.08E-20 | 3.86777E-19 | RNA polymerase subunit sigma-70 |
| WP_060383508.1 | 1.7071 | 7.135941 | 5.87E-20 | 1.91812E-18 | hypothetical protein |
| WP_060383674.1 | 1.71309 | 12.90898 | 2.25E-14 | 4.63631E-13 | X-Pro aminopeptidase |
| WP_060383035.1 | 1.71592 | 6.557008 | 3.29E-12 | 4.84555E-11 | T9SS C-terminal target domain-containing protein |
| WP_060382629.1 | 1.72352 | 9.025028 | 2.02E-28 | 1.33609E-26 | hypothetical protein |
| WP_060381305.1 | 1.73328 | 8.374591 | 7.84E-24 | 3.7199E-22 | glycosyl transferase family 1 |
| WP_060382068.1 | 1.73953 | 7.5756 | 1.20E-27 | 7.3874E-26 | glutathionylspermidine synthase |
| WP_060383176.1 | 1.7465 | 5.763719 | 2.75E-17 | 7.06034E-16 | hypothetical protein |
| WP_060381970.1 | 1.74755 | 6.443583 | 1.19E-27 | 7.3874E-26 | organic hydroperoxide resistance protein |
| WP_060381372.1 | 1.74968 | 9.168523 | 2.21E-17 | 5.73875E-16 | chromosome partitioning protein |
| WP_060382235.1 | 1.75355 | 4.935591 | 2.36E-18 | 6.73394E-17 | cytochrome-c peroxidase |
| WP_060383668.1 | 1.7578 | 7.869841 | 5.77E-19 | 1.76893E-17 | thioredoxin |
| WP_060382004.1 | 1.79688 | 9.591382 | 1.26E-07 | 7.68007E-07 | acetyl-CoA acetyltransferase |
| WP_060382066.1 | 1.80665 | 3.958931 | 2.87E-23 | 1.28859E-21 | hypothetical protein |
| WP_060381444.1 | 1.81865 | 10.16969 | 4.36E-12 | 6.26213E-11 | hypothetical protein |
| WP_060381362.1 | 1.84503 | 6.06762 | 3.44E-24 | 1.66267E-22 | secretion protein |
| WP_060382095.1 | 1.84563 | 5.622364 | 3.90E-25 | 2.0013E-23 | MarR family transcriptional regulator |
| WP_060381538.1 | 1.84568 | 11.62834 | 1.35E-10 | 1.52806E-09 | hypothetical protein |
| WP_060382002.1 | 1.85092 | 10.43465 | 2.02E-12 | 3.09747E-11 | acyl-CoA dehydrogenase |
| WP_060382870.1 | 1.85488 | 10.4933 | 9.83E-15 | 2.11434E-13 | esterase |
| WP_014165082.1 | 1.87258 | 9.468887 | 1.85E-11 | 2.3555E-10 | DNA-binding protein |
| WP_060382464.1 | 1.87375 | 7.73979 | 5.48E-17 | 1.3517E-15 | TonB-dependent receptor |
| WP_060382447.1 | 1.89805 | 8.059357 | 2.06E-31 | 1.62101E-29 | hypothetical protein |
| WP_060383327.1 | 1.90726 | 10.64187 | 5.37E-12 | 7.58534E-11 | electron transfer flavoprotein subunit alpha |
| WP_060381373.1 | 1.92936 | 7.109908 | 2.61E-23 | 1.1939E-21 | hypothetical protein |
| WP_060381689.1 | 1.9313 | 10.37171 | 9.56E-12 | 1.27962E-10 | flagellar motor protein MotA |
| WP_060382549.1 | 1.93334 | 7.602577 | 5.79E-18 | 1.58313E-16 | FAD-dependent oxidoreductase |
| WP_060381306.1 | 1.94341 | 8.608299 | 7.44E-23 | 3.11876E-21 | hypothetical protein |
| WP_060383433.1 | 1.95218 | 10.60813 | 4.58E-20 | 1.53522E-18 | efflux transporter periplasmic adaptor subunit |
| WP_060381980.1 | 1.96606 | 2.901708 | 1.75E-07 | 1.03978E-06 | ArsR family transcriptional regulator |
| WP_060381901.1 | 1.98371 | 7.581453 | 1.16E-18 | 3.46512E-17 | hypothetical protein |
| WP_060383412.1 | 1.9903 | 11.59059 | 1.85E-22 | 7.5227E-21 | signal peptide peptidase SppA |
| WP_060383037.1 | 1.99687 | 11.24629 | 7.62E-11 | 8.9537E-10 | hypothetical protein |
| WP_060382829.1 | 2.00139 | 8.49837 | 1.55E-25 | 8.13333E-24 | hypothetical protein |
| WP_060381920.1 | 2.02304 | 7.05944 | 5.08E-25 | 2.5541E-23 | abortive phage infection protein |
| WP_060382941.1 | 2.02749 | 7.16821 | 1.04E-14 | 2.20905E-13 | hypothetical protein |
| WP_060381836.1 | 2.02881 | 3.540121 | 8.93E-14 | 1.65254E-12 | UDP-N-acetylmuramate--alanine ligase |
| WP_060383401.1 | 2.03481 | 9.349299 | 3.24E-19 | 1.01952E-17 | hypothetical protein |
| WP_060383661.1 | 2.07266 | 6.832323 | 7.43E-20 | 2.36759E-18 | patatin |
| WP_060381668.1 | 2.08939 | 7.619535 | 9.51E-12 | 1.27925E-10 | 2-nitropropane dioxygenase |
| WP_060382614.1 | 2.10649 | 3.763124 | 4.36E-19 | 1.35291E-17 | hypothetical protein |
| WP_060382135.1 | 2.12031 | 5.208551 | 2.20E-23 | 1.02566E-21 | hypothetical protein |
| WP_060381615.1 | 2.1454 | 8.624158 | 5.23E-25 | 2.5801E-23 | hypothetical protein |
| WP_060382034.1 | 2.15894 | 8.161425 | 2.74E-15 | 6.16E-14 | hypothetical protein |
| WP_060382395.1 | 2.16363 | 4.718922 | 1.10E-13 | 1.99711E-12 | hypothetical protein |
| WP_014164868.1 | 2.20805 | 11.06844 | 2.74E-18 | 7.6507E-17 | RNA polymerase sigma factor RpoD |
| WP_060381632.1 | 2.22442 | 8.668103 | 1.26E-20 | 4.41003E-19 | hypothetical protein |
| WP_060382125.1 | 2.23533 | 2.253881 | 5.51E-12 | 7.70354E-11 | hypothetical protein |
| WP_060382504.1 | 2.27137 | 5.862975 | 2.30E-12 | 3.48314E-11 | hypothetical protein |
| WP_060381967.1 | 2.2968 | 6.314766 | 1.11E-37 | 1.27042E-35 | hypothetical protein |
| WP_060382136.1 | 2.30005 | 4.140716 | 5.80E-21 | 2.17734E-19 | hypothetical protein |
| WP_060381366.1 | 2.31063 | 8.277305 | 1.22E-15 | 2.80777E-14 | histidine kinase |
| WP_060382550.1 | 2.32598 | 8.998759 | 9.88E-21 | 3.60354E-19 | glycerol kinase |
| WP_060382624.1 | 2.35594 | 8.6717 | 5.92E-26 | 3.23622E-24 | hypothetical protein |
| WP_060383434.1 | 2.35604 | 9.679931 | 5.07E-22 | 1.9938E-20 | transporter |
| WP_060381974.1 | 2.37387 | 4.697825 | 8.15E-14 | 1.51832E-12 | catalase |
| WP_060382699.1 | 2.40734 | 12.33655 | 1.17E-20 | 4.15123E-19 | peptidase M16 |
| WP_060382005.1 | 2.43096 | 10.94988 | 2.84E-14 | 5.77248E-13 | 3-hydroxyacyl-CoA dehydrogenase |
| WP_060382994.1 | 2.43579 | 8.530702 | 7.12E-38 | 8.53541E-36 | LemA family protein |
| WP_060383098.1 | 2.45416 | 7.473981 | 2.44E-18 | 6.89944E-17 | hypothetical protein |
| WP_060382465.1 | 2.48053 | 7.017035 | 8.81E-18 | 2.38363E-16 | deoxyribonuclease HsdR |
| WP_060383795.1 | 2.49603 | 8.877857 | 4.75E-26 | 2.65594E-24 | RNA-splicing ligase RtcB |
| WP_060383657.1 | 2.5114 | 5.62707 | 1.32E-15 | 3.02623E-14 | iron transporter |
| WP_060382622.1 | 2.54561 | 10.19297 | 1.31E-29 | 9.15757E-28 | TonB-dependent receptor |
| WP_060382448.1 | 2.54709 | 7.458086 | 7.64E-37 | 8.00583E-35 | DNA starvation/stationary phase protection protein |
| WP_060383786.1 | 2.63472 | 11.71884 | 1.36E-28 | 9.2428E-27 | endothelin-converting protein |
| WP_060382995.1 | 2.64056 | 1.310361 | 2.69E-13 | 4.51354E-12 | hypothetical protein |
| WP_060382069.1 | 2.69478 | 10.6691 | 1.73E-27 | 1.03677E-25 | hypothetical protein |
| WP_060382466.1 | 2.75009 | 8.818583 | 4.43E-23 | 1.8898E-21 | hypothetical protein |
| WP_060381532.1 | 2.79008 | 7.185773 | 7.18E-39 | 9.51113E-37 | enoyl-CoA hydratase |
| WP_060382588.1 | 2.81988 | 9.476391 | 9.73E-21 | 3.60068E-19 | phosphate transport regulator |
| WP_060381835.1 | 2.94721 | 3.316255 | 1.08E-35 | 1.04178E-33 | hypothetical protein |
| WP_060383353.1 | 3.06107 | 9.133181 | 2.04E-41 | 3.20594E-39 | ABC transporter permease |
| WP_060383004.1 | 3.07111 | 3.757444 | 5.97E-14 | 1.15455E-12 | sphingomyelinase/phospholipase |
| WP_060381307.1 | 3.19334 | 8.183417 | 3.84E-23 | 1.66776E-21 | contain sulphate transporter domian |
| WP_060381365.1 | 3.34264 | 9.564651 | 6.73E-30 | 4.83873E-28 | ArsR family transcriptional regulator |
| WP_060383099.1 | 3.52502 | 6.906051 | 4.09E-30 | 3.02981E-28 | Flagelllin biosynthesis protein |
| WP_060383191.1 | 3.57809 | 8.102602 | 3.60E-26 | 2.05761E-24 | export.efflux prptein |
| WP_060382467.1 | 3.57947 | 11.30332 | 3.26E-26 | 1.90526E-24 | Hypothetical protein |
| WP_060382672.1 | 3.63817 | 6.791994 | 3.55E-30 | 2.70728E-28 | thioesterase protein |
| WP_060382673.1 | 3.64058 | 9.88809 | 5.96E-20 | 1.92267E-18 | Hypothetical protein |
| WP_060382698.1 | 3.66875 | 9.206716 | 1.13E-36 | 1.13348E-34 | Phosphate ABC transporter protein |
| WP_060382674.1 | 3.79544 | 9.665114 | 1.25E-25 | 6.68137E-24 | Hypothetical gene |
| WP_060382006.1 | 3.8101 | 7.052608 | 2.83E-20 | 9.62435E-19 | MarR family transcriptional regulation |
| WP_060382096.1 | 3.81795 | 9.91266 | 1.20E-41 | 2.01E-39 | Band 7 protein. Integral membrane protein |
| WP_060381858.1 | 3.87902 | 6.351945 | 3.56E-28 | 2.29683E-26 | secreted phosphatase, PhoX family |
| WP_060382677.1 | 3.87935 | 9.86124 | 9.63E-32 | 8.36E-30 | lipoprotein |
| WP_060382676.1 | 4.08411 | 12.02067 | 1.13E-31 | 9.17136E-30 | Hypothetical protein. Domain: transcriptional regulator |
| WP_060383100.1 | 4.11384 | 8.472096 | 7.55E-34 | 7.0381E-32 | Hypothetical protein. |
| WP_060382675.1 | 4.24408 | 9.826139 | 9.14E-32 | 8.21E-30 | PadR family transcriptional regulator repressors |
| WP_060383185.1 | 4.38745 | 6.20772 | 2.84E-37 | 3.11E-35 | LuxR family trascriptional regulator |
| WP_060382775.1 | 4.53702 | 10.66737 | 1.03E-39 | 1.44034E-37 | Ton-B dependent receptor |
| WP_060383183.1 | 4.66217 | 8.909717 | 4.28E-38 | 5.38476E-36 | peptidase M4 |
| WP_060383184.1 | 5.13643 | 6.494663 | 2.28E-44 | 4.41475E-42 | hypothetical protein |
| WP_060383101.1 | 5.78115 | 11.78905 | 2.62E-41 | 3.87554E-39 | Ton-B dependent receptor |
| WP_060381364.1 | 5.78782 | 10.49137 | 6.40E-60 | 2.68351E-57 | Ton-B dependent receptor |
| WP_060383190.1 | 5.83854 | 8.853831 | 2.72E-49 | 7.59126E-47 | siderophore biosynthesis protein lucA |
| WP_060381363.1 | 6.05667 | 9.930441 | 6.38E-63 | 5.35284E-60 | hypothetical protein |
| WP_060383182.1 | 6.42899 | 11.59184 | 1.29E-56 | 4.05393E-54 | TonB dependent receptor |
| WP_060383861.1 | 6.61798 | 10.03701 | 6.75E-48 | 1.69856E-45 | TonB dependent or siderophore |
| WP_060382681.1 | 7.08582 | 11.17095 | 3.02E-62 | 1.9018E-59 | hypothetical protein |
| WP_060383888.1 | 7.33521 | 9.176353 | 1.39E-65 | 1.75289E-62 | siderophore (alcaligin) biosythesis |
| WP_060382680.1 | 7.61292 | 10.54294 | 2.43E-69 | 6.11844E-66 | HmuY Protein |
| WP_060382679.1 | 7.77678 | 11.59612 | 1.77E-46 | 4.0506E-44 | Secretion protein |
| WP_060383188.1 | 8.07646 | 9.760374 | 3.97E-44 | 7.14045E-42 | DUF1624 domain-containing protein |
| WP_060383189.1 | 8.18876 | 10.96539 | 1.06E-57 | 3.81349E-55 | transcriptional regulator |
| WP_060383186.1 | 8.24561 | 10.58058 | 2.18E-60 | 1.0989E-57 | aspartate aminotransferase family protein |
| WP_060383187.1 | 8.45426 | 10.87413 | 3.82E-45 | 8.00051E-43 | siderophore alcaligin biosynthesis protein |
