# Supplementary material for: Transcriptome analysis of the fish pathogen Flavobacterium columnare in biofilm suggests calcium role in pathogenesis
Source: BMC Microbiol. 2019 Jul 4;19:151. doi: 10.1186/s12866-019-1533-4 (PMC6610971; doi:10.1186/s12866-019-1533-4)
Supplement: Supplementary file 2 — Supplemental Table 4. Primers used for qPCR. Figure S1. Library size for all samples. Figure S2. Heat-map of the top-50 DEGs expression profiles among the 9 samples after 48 h incubation. Figure S3. Gene ontology annotation of the up- (panel A) and down-regulated (panel B) genes between samples. (DOCX 992 kb) [file 12866_2019_1533_MOESM2_ESM.docx]

**Table S1.** Primers used for qPCR validation (5’ to 3’)

| Gene | Forward | Reverse |
| --- | --- | --- |
| Siderophore | TCAGAGCGCAGCAGAAGTTT | TGATCTCGTTTGGCTTCTGGT |
| T9SS | AATGGAGCTTGGAAAGGGAA | TGAGGCTCTTCATCCACAACA |
| TonB-dependent receptor | TTCATTACAGCCCAGCGGTT | TTAGCCCCATGCGTAACACC |
| NADH: ubiquinone oxidoreductase subunit H | ATTGCCAA CAGCAACAGG | GGTGACCACCAATAAGTTCAGC |
| Catalase | TATTGACGGTAACGGCGGAG | AGGTTACCTGGTTGTGTGTAGT |
| Quorum sensing | ACCTTTCGCACACACAGAAA | GCAATGTCGTTCTTTAGGCTGT |
| Cytochrome c oxidase accessory protein | ACAGTCTCCCTTGCCTAATAAAGA | AGGACCACTACACAATACAGGT |
| Protein-tryosine-phosphatase | AGGACATCCCTCATCTGCATT | TCTGGAGGAACAGAAAGTACCAC |
| Adenylate kinase (reference gene) | TTCTTTTCAACTTCAGATTCCAACA | GGAAAACCTGGAGCGGGAA |


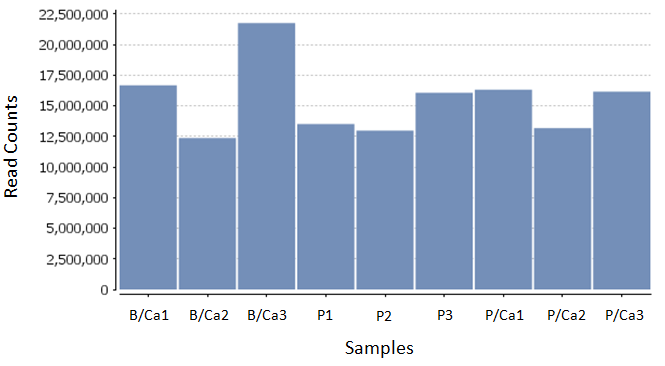


**Figure S1.** Library size that were mapped to the reference coding sequences. B/Ca represents biofilm cells in 4.5 mM [Ca2+]; P, represents planktonic cells in control medium, P/Ca represents planktonic cells in 4.5 mM [Ca2+].


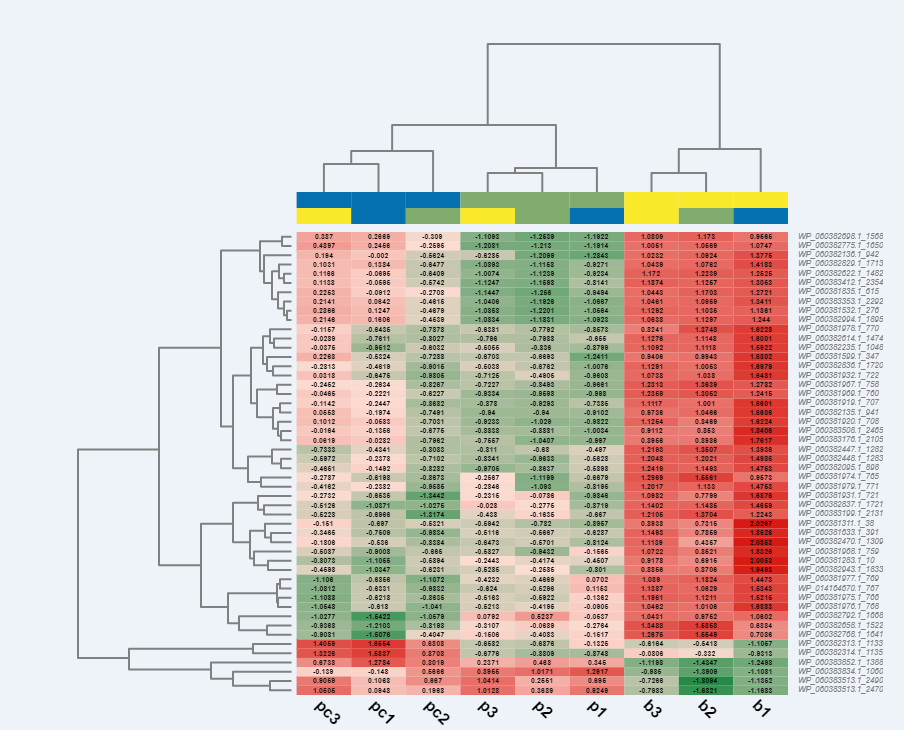


P/C3

P/C1

P/C2

P3

P2

P1

B/Ca3

B/Ca2

B/Ca1

**Figure S2.** Heat-map of the top-50 differentially expressed genes expression profiles among the 9 samples after 48 h incubation. Red and green colors indicate up- and down-regulation as log2 transformed values, respectively. P resents planktonic samples in control medium; pc represents planktonic samples in calcium-supplemented medium; bc represents biofilm samples in calcium-supplemented medium.


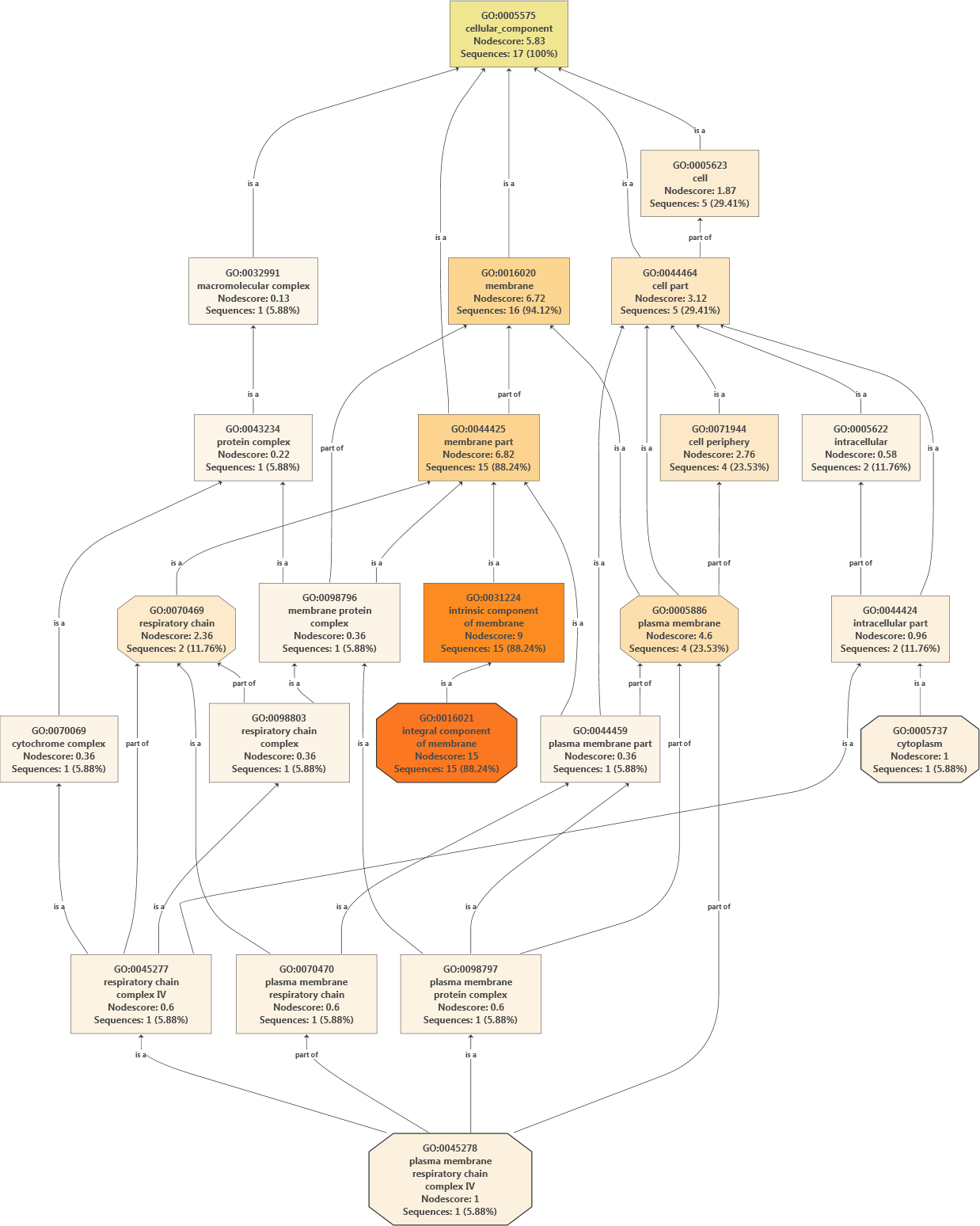


A

**
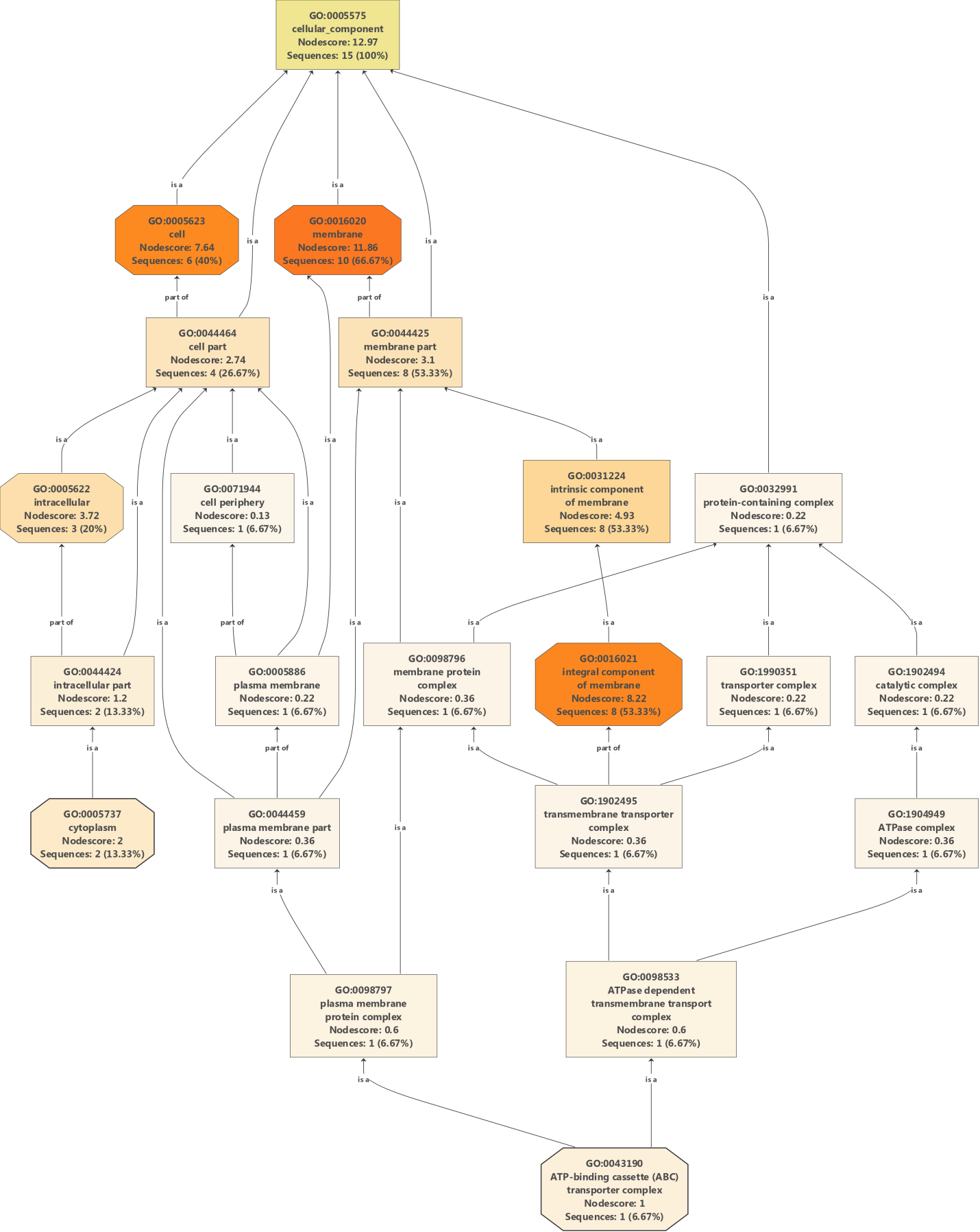
**

**Figure S3.** GO annotation of the up- (panel A) and down- (panel B) regulated genes between biofilm and planktonic samples in Ca-supplemented medium.

B

B
